# In Situ TEM Imaging Reveals the Dynamic Interplay Between Attraction, Repulsion and Sequential Attraction‐Repulsion in Gold Nanoparticles

**DOI:** 10.1002/smll.202406943

**Published:** 2024-10-08

**Authors:** Abid Zulfiqar, Mari Honkanen, Nonappa, Minnamari Vippola

**Affiliations:** ^1^ Faculty of Engineering and Natural Sciences Tampere University Tampere FI‐33720 Finland; ^2^ Tampere Microscopy Center Tampere University Tampere FI‐33720 Finland

**Keywords:** coalescence, in situ TEM, nanoparticle attraction and repulsion, nanoparticle manipulation, nanoscale dynamics, structural transformation

## Abstract

Recent efforts on manipulating metal nanoparticles (NPs) using an electron beam have offered new insights into nanoparticle behavior, structural transition, and the emergence of new properties. Despite an increasing understanding of the dynamics of electron beam‐induced coalescence of NPs, several phenomena are yet to be investigated. Here, we show that repulsion between two NPs is as favorable as coalescence under electron beam irradiation at room temperature. Using small‐sized (*D* ≈ 5.9 nm) and large‐sized (*D* ≈ 11.0 nm) gold (Au) NPs, and different electron dose rates, a unique sequential attraction‐repulsion between NPs is disclosed. The real‐time in situ transmission electron microscopy imaging suggest that at a low dose rate, two small‐sized AuNPs with 1.0 nm particle–particle distance undergo repulsion to 18 nm with a diffusion rate of 0.4 nm min^−1^. For large‐sized AuNPs, the repulsion rate is 0.08 nm min^−1^ at a low dose rate and is comparable to that of small‐sized AuNPs at a high dose rate. Surprisingly, large‐sized AuNPs at a high electron dose rate displayed attraction in the first 15 min, followed by rapid repulsion. This unique sequential attraction‐repulsion behavior of NPs offers possibilities to manipulate interparticle distance and properties without inducing dimensional changes for advanced photonic and plasmonic nanodevices.

## Graphical abstract

The effect of electron beam dose rate, nanoparticle size, and interparticle distance on the in situ nanoscale dynamics of gold nanoparticles are investigated. The results revealed that two other dynamic processes, repulsion, and sequential attraction‐repulsion are also as favorable as that of routinely observed coalescence between two gold nanoparticles.

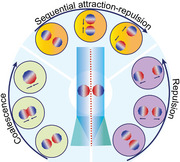

## 1 Introduction

Understanding the real‐time nanoscale dynamics is crucial to precisely manipulating the structure, mechanical performance, and optoelectronic properties of metal nanoparticles (NPs).^[^ ^1^ ^]^ In recent years, considerable efforts have been made to study the behavior of metal NPs under mechanical compression,^[^ ^2^ ^]^ thermal treatment,^[^ ^3^ ^]^ and electron beam irradiation.^[^ ^4^, ^5^ ^]^ Importantly, transmission electron microscopy (TEM) and scanning transmission electron microscopy (STEM) imaging allow real‐time visualization of electron beam irradiation‐induced dynamics of gold (Au), silver (Ag), palladium (Pd) and bimetallic NPs.^[^ ^6^, ^7^, ^8^ ^]^ The high energy electron beam results in ionization, charging, phase transition, and beam‐induced defect resulting in structural change, including twinning and detwinning of nanomaterials under study.^[^ ^9^, ^10^, ^11^, ^12^, ^13^ ^]^ Controlling the electron beam dose rate, exposure time, temperature and substrate allows careful manipulation of individual NPs, nanoparticle (NP) pairs, assemblies, and even in situ TEM‐assisted synthesis of NPs from solid precursors.^[^ ^14^, ^15^, ^16^, ^17^ ^]^ Among structural transformation, electron beam irradiation and temperature‐induced coalescence an extensively studied phenomena in metal NPs.^[^ ^18^, ^19^, ^20^ ^]^ When two spatially isolated but closely placed particles or particles in mutual contact are irradiated with an electron beam, it results in a structural or dimensional change depending on the particle size, interparticle distance *d_r_*, and interfacial adhesion (i.e., how strongly the particles have adhered to the surface) due to strong coalescence tendency.^[^ ^4^ ^]^ The driving force for coalescence is the reduction in the surface energy of a newly formed structure.^[^ ^21^ ^]^ The reduction in surface energy is facilitated by the mobility of surface atoms or particle diffusion and the reorganization by orientational alignment of coalescing lattice planes at the interface of the coalescing particles. Therefore, coalescence is affected by the size of the NPs, electron beam dose rate, diffusion coefficient *C_d_*, and interfacial adhesion. The diffusion of NPs is strongly influenced by the NP size due to the inverse relation of *C* *_d_* with the dimension *D* of the NP as *C_d_∝ D* ^−(1‐3α/α)^.^[^ ^4^ ^]^ According to von Smoluchowski´s kinetic equation, the diffusion depends on the particle *D* and time (*t*) as *D ∝ t^−^* ^α^, where *α* is a constant depending on the strength of interfacial adhesion.^[^ ^4^ ^]^

José‐Yacamán et al. demonstrated that under electron beam irradiation small PdNPs (*D* ≈ 3 nm) undergo coalescence into a truncated octahedron.^[^ ^4^ ^]^ On the other hand, larg bimetallic Au‐PdNPs (*D* ≈ 11 nm) resulted in partial coalescence. Furthermore, when two different‐sized AuNPs were used, the small particles moved toward large particles, leading to coalescence. Cheng et al. studied the nano curvature effect and electron beam athermal activation effect in the coalescence of AuNPs.^[^ ^18^ ^]^ Their study revelead the coalescence of single crystalline AuNPs on amorphous silicon oxide (SiOx) substrate under electron beam irradiation at room temperature. Lim et al. reported the real‐time imaging and kinetic Monte Carlo continuum simulations of decahedral AuNPs.^[^ ^22^ ^]^ Their study indicated that clear lattice fringes were visible throughout TEM observations, ruling out any melting or liquid phase  during coalescence. Yuk et al. utilized graphene‐supporting membranes to study neck formation, oriented attachment, and structural relaxation of AuNPs.^[^ ^23^ ^]^ In another study, Wang et al. argued that thermodynamic fluctuations and atomic interaction forces induced the coalescence of AuNPs on silicon surfaces.^[^ ^20^ ^]^ Baston et al. showed that the two particles can be pushed apart by selectively placing the electron beam between a particle pair. It was also revealed that when an electron beam was placed near individual NPs, they showed long‐range attractive and short‐range repulsive forces.^[^ ^24^ ^]^

In recent years, tremendous progress has been made using experimental research and simulations on the coalescence of NPs under electron beam irradiation. However, systematic studies on repulsion or attraction between NPs using conventional electron beam irradiation are limited in the literature. Identifying and understanding the parameters to control repulsion or attraction will offer methods to overcome the coalescence or structural transition of two closely placed NPs. By preventing unwanted structural transition, such methods will pave the way for precise manipulation of *d_r_* for advanced photonic and plasmonic nanodevices. In this work, we reveal that under identical experimental conditions, unlike the widely studied structural transformation and coalescence of AuNPs, repulsion, and sequential attraction‐repulsion between NPs are as favorable as coalescence. Using two differently sized AuNPs *viz*., small‐sized (*D* ≈ 5.9 nm) and large‐sized (*D* ≈ 11 nm), we studied: i) structural transition of individual NPs, ii) coalescence between NPs, iii) repulsion between NPs and iv) sequential attraction‐repulsion between NPs. We demonstrate the effect of electron beam dose rates, size of individual AuNPs, *d_r_*, and difference in particle sizes on structural transition, coalescence, repulsion, and sequential attraction‐repulsion behavior.

## 2 Results and Discussion

### 2.1 Characterization of Gold (Au) Nanoparticles (NPs)

**Figure 1:**
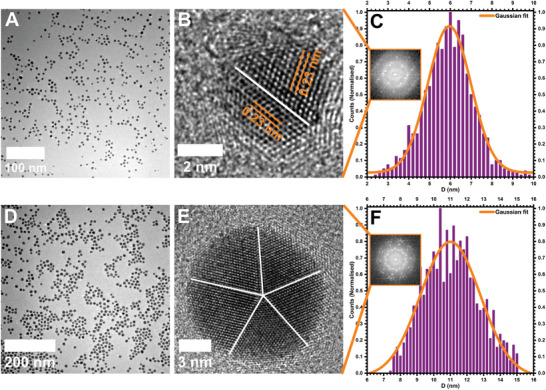
Transmission electron microscopy (TEM) imaging of small‐sized (top row) and large‐sized (bottom row) gold (Au) nanoparticles (NPs). A) Low magnification TEM image of small‐sized AuNPs. B) High resolution (HR)‐TEM image of a single small‐sized AuNP showing fcc lattice with a single twin boundary. C) Histogram showing the normalized size distribution of small‐sized AuNPs with a bin width of 0.2 nm. D) Low magnification TEM image of large‐sized AuNPs. E) HR‐TEM image of a single large‐sized AuNP showing the five‐fold symmetry of the decahedral structure. F) Histogram showing the normalized size distribution of large‐sized AuNPs with a bin width of 0.2 nm. The insets in (C and F) show the corresponding FFT patterns of HR‐TEM images in (B and E).

We have used the AuNPs stabilized in citrate buffer obtained from commercial sources (see details in the experimental section). The morphologies and size distribution analysis of small and large‐sized AuNPs were performed using TEM imaging (**Figure** **1**A,D). The high‐resolution TEM (HR‐TEM) images of individual NPs revealed a face‐centered cubic (fcc) structure, confining a single twin boundary with lattice fringes (*d* ≈ 0.23 nm) corresponding to the (111) plane of Au in small‐sized AuNPs (Figure 1B), and decahedral structure with fivefold symmetry of the twin boundaries in large‐sized AuNPs (Figure 1E). The presence of decahedral morphology with a five‐fold twin structure in large‐sized AuNPs and other metal NPs of similar size, such as AgNPs has been widely studied in the literature and is considered a stable morphology at room temperature due to their low surface anisotropy and low twinning energies.^[^ ^21^, ^25^, ^26^, ^27^ ^]^

The size distribution analysis based on the TEM images showed a Gaussian distribution centered at 5.9 ± 1.2 nm and 11.0 ± 1.6 nm, respectively for small and large‐sized AuNPs (Figure 1C,F). After morphological characterization, we used these AuNPs for in situ TEM observations of the dynamic behavior under continuous electron beam irradiation. Specifically, we focused on the effect of NP size, *d_r_* (edge‐edge), and electron dose rate on the coalescence, attractive, and repulsive behavior between spatially isolated AuNPs.

### 2.2 Effect of the Electron Beam on Individual AuNPs

**Figure 2:**
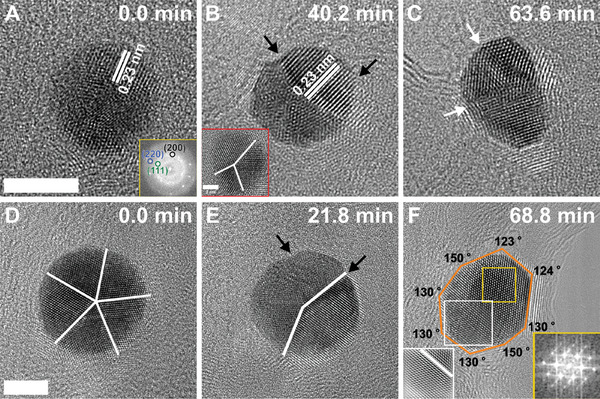
Effect of electron beam dose on individual NPs. Selected HR‐TEM images showing the structural transition of a small‐sized AuNP A–C) and a large‐sized AuNP D–F) under the continuous exposure of the electron beam. The yellow insets in (A and F) show the corresponding FFT patterns, while the red inset in (B) displays the twinned particle at 15 min. The white inset presents the inverse FFT image marked in (F), showing the location of the twin boundary. The black arrows show the formation of surface facets, whereas the white arrows and lines mark the location of the twin boundaries. The scale bar is 5 nm, and the electron beam dose rate is 1.2 × 10^5^ e^−^ Å^−^^2^s^−1^.

In electron microscopy imaging, the interaction of the electron beam with the NP leads to elastic and inelastic scattering of the electrons.^[^ ^28^ ^]^ The high‐energy electrons can transfer the energy to atoms within the NPs, resulting in radiation damage and structural transformation. Under certain conditions, the elastic scattering can result in electrostatic charging, atom displacement, or electron beam sputtering of surface atoms.^[^ ^9^ ^]^ Similarly, inelastic scattering results in specimen heating or radiolysis, inducing structural damage and mass loss of the materials. However, the radiation damage depends on the electron beam dose, exposure time, and the material under investigation. To gain insight into the structural and shape transformation of individual AuNPs, TEM imaging was performed upon continuous exposure to the electron beam for more than 1 h. In a small‐sized AuNP, the electron beam transformed the icosahedral particle (**Figure** **2**A) to a twinned particle in ≈15 min when a dose rate of 1.2 × 10^5^ e^−^ Å^−^^2^s^−1^ was used (inset of Figure 2B). This twinned structure further transformed into a decahedral particle with prominent surface facets after 40 min of irradiation (Figure 2B). The structural transformation of small‐sized AuNPs observed in this work aligns with previous reports on the transformation of an icosahedral to a decahedral structure at elevated temperatures.^[^ ^25^, ^29^ ^]^ However, the current findings indicate that such a transition is also favorable at room temperature when the NPs are irradiated with an electron beam. Further exposure resulted in a change from the spherical shape to the elongation of the NP with twin boundaries. This is attributed to the continuous diffusion and redistribution of atomic density for the formation of new lattice fringes to acquire a stable morphology (Figure 2C). More importantly, the surface facets remained intact despite the elongation of AuNP, suggesting a knocking mechanism upon the interaction with an electron beam.

Similarly, for a large‐sized AuNP, the continuous exposure of an electron beam initially transforms the decahedral particle with a five‐fold twin symmetry parallel to the electron beam, to a decahedral facet structure at *t* = 22 min (Figure 2D,E). The structural transformation in a five‐fold twinned symmetry involves a detwinning mechanism by symmetry breaking of the twin structure (Figure 2D).^[^ ^12^ ^]^

Due to unbalanced strain distribution with the formation of new surfaces, twin interfaces migrate from the center to the periphery region via surface diffusion of Au atoms (Figure 2E).^[^ ^12^, ^13^ ^]^ It has been reported in the literature that the detwinning process of a fivefold crystal can be initiated by phase transformation,^[^ ^30^ ^]^ atomic diffusion,^[^ ^31^ ^]^ dislocation density, and twin interface migration due to strain relaxation.^[^ ^12^ ^]^ Furthermore, time‐resolved HR‐TEM imaging technique and molecular dynamics simulations have shown that the migration of twin boundaries is facilitated via partial dislocation slipping under an electron probe to reduce the total surface energy.^[^ ^12^, ^13^, ^32^, ^33^ ^]^ Therefore, the breaking of twin symmetry induced by strain relaxation evolved the more homogenous morphology, showing an approximately truncated octahedron structure, with a single twin boundary (Figure 2F). The corresponding selected FFT pattern in the inset shows the structural transformation with crystallographic orientation and planes for a more favorable stable structure at *t* = 69 min.

### 2.3 Coalescence in AuNPs

**Figure 3:**
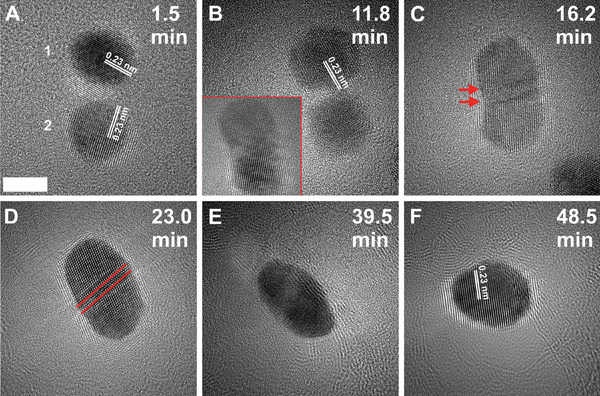
TEM micrographs showing the coalescence of small‐sized AuNPs. A) Two AuNPs with an interparticle distance *d_r_* (edge‐edge) of 1.3 nm with observed diameters of *D_1_* = 7.4 nm and *D_2_* = 7.1 nm for NPs 1 and 2, respectively at 1.5 min of electron beam irradiation. B) At 12.0 min a neck connection is established between two NPs. C,D) The formation of a rod‐like structure after the coalescence with internal stresses where red arrows and lines reveal the location of twin boundaries in the coalesced structure. E,F) The continuous irradiation transforms the structure closely to a spherical shape with the change in atomic contrast. The inset in (B) shows the coalesced structure at 14.2 min. The scale bar is 5 nm, and the dose rate is 3 × 10^4^ e^−^ Å^−^^2^s^−1^.

The coalescence in NPs was studied for small and large‐sized AuNPs based on the appropriate distance between the closely placed AuNPs. First, we discuss the coalescence behavior of small‐sized AuNPs. **Figure** **3**A–F shows the selected TEM images of the coalescence of a pair of small‐sized AuNPs upon increasing exposure to an electron beam with a constant electron dose rate of 3 × 10^4^ e^−^ Å^−^^2^s^−1^ (see Supporting Information for an explanation on determining electron beam dose rate). The two AuNPs of diameters 7.4 nm (*D*_1_) and 7.1 nm (*D*_2_) were initially located at a *d*_r_ of 1.3 nm. The observed diameters were somewhat larger than the average size of small‐sized AuNPs. However, they are within the given window of small‐sized AuNPs size distribution (Figure 1C). Figure 3A shows the overview of two NPs before the coalescence after the illumination of the electron beam at time *t* = 1.5 min (i.e., the electron beam illumination time), where *d_r_* was unchanged (hence TEM image at *t* = 0 min is not given here). The continued irradiation promoted the diffusion of AuNPs across the surface for coalescence toward each other (Movie S1, Supporting Information). Such a process can be attributed to the coupled plasmon modes, which minimize the overall surface energy due to attractive interparticle forces.^[^ ^6^, ^34^, ^35^ ^]^ The diffusion of NPs is attributed to their weak adhesion with the carbon surface,^[^ ^4^ ^]^ where the interaction between the two coupled NPs under the electron beam irradiation generates more localized plasmons between NPs. This results in the hybridization of the individual particle modes and enhances the electromagnetic field at the interparticle junction depending upon the *d_r_*, NP size and electron dose.^[^ ^36^, ^37^, ^38^ ^]^ Here, it is important to note that the coalescence mechanism of AuNPs in solution, surfaces, and under various imaging conditions is well documented in the literature.^[^ ^4^, ^20^, ^22^, ^39^ ^]^

Briefly, upon the irradiation of an electron beam, the two NPs approached each other for coalescence with a neck‐like structure formation within a *t* of 11.8 min (Figure 3B). Before establishing a neck connection, both NPs rotated to align their lattice planes in the same orientation with respect to each other without changing their lattice spacings (*d* ≈ 0.23 nm) using the oriented attachment route. Here, it is interesting to note that the lower NP was rotated away from a zone axis just before forming the neck connection (Figure 3B), which later changed its crystallographic orientation upon neck formation at 14.2 min (inset of Figure 3B). The lattice realignment may favor the coalescence mechanism,^[^ ^5^, ^39^ ^]^ and, therefore, takes a longer time to establish a neck connection with each other under electron beam irradiation. Surface diffusion plays a crucial role in the initial stage of coalescence when two particles come in contact, driven by the reduction of surface energy via the diffusion of atoms on the surfaces across the interface. This led to the formation of a neck connection and induced the movement of atoms between the two NPs, growing neck and reorganizing the whole structure (Figure 3C).

Figure 3D shows the TEM image of structural reorganization or reconstruction of the surface after *t* = 23 min. The continuous growth of the neck imparts significant local stress and strain at the interface, inducing two parallel twin boundaries (indicated with red lines in Figure 3D) to accommodate the strain for reducing the overall surface energy. In situ TEM observations showed that these twin boundaries were structured at the interface by the crystallographic rotation of the lower part of the coalesced structure (inset in Figure 3B; Movie S1, Supporting Information). The formation of structural defects such as twin boundaries, during coalescence, has widely been investigated in the literature.^[^ ^4^, ^23^, ^40^ ^]^ The emergence of twin boundaries enhances the internal stresses, leading to instability of the structure. Nonetheless, the continuous exposure of the electron beam facilitated movement and eventual elimination of structural defects such as twin boundaries. The atomic diffusion toward the center of the coalesced structure was observed with the change in atomic contrast (Figure 3E).

The structure was further rearranged to a nearly spherical shape single crystal structure with approximately *d* = 0.24 nm at *t* = 48.5 min (Figure 3F; Movie S1, Supporting Information). The change in the shape of the structure is governed by the diffusion and redistribution of atoms in the whole structure. This results in the formation of new surfaces and reconstruction of the structure while minimizing the internal stresses and surface energy in the new structure.^[^ ^41^ ^]^ Therefore, the final structure is referred to as a reconstructed structure (Figure 3E,F; Movie S1, Supporting Information).

**Figure 4:**
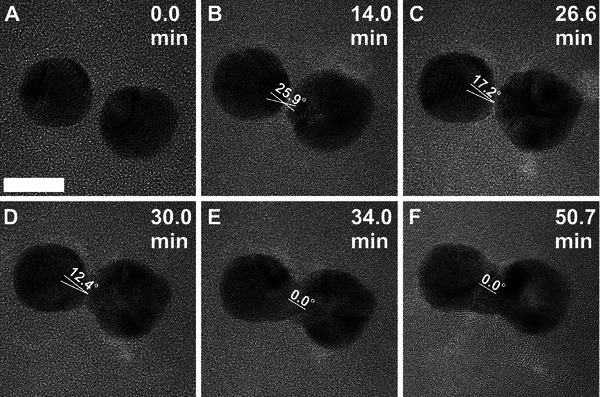
A series of TEM micrographs showing the coalescence of large‐sized AuNPs. A) TEM image of two AuNPs with a *d*_r_ of 1.4 nm at *t* = 0. B) NPs approach each other for coalescence. C) Misalignment of lattice planes for neck formation. D,E) Growth of neck diameter *D_n_* and change in atomic contrast. F) Formation of a dumbbell‐like rod‐shaped structure. The *t* is given on the top right corners of each sequential frame. The length of the scale bar is 10 nm, and the dose rate is 6.6 × 10^4^ e^−^ Å^−^^2^s^−1^.

Under similar experimental conditions, the coalescence of large‐sized AuNPs occurred through a twin boundary formation at the interface region. This is due to the mismatching of lattice planes between two NPs, which is attributed to NPs´ stability caused by lower surface energy compared to small‐sized NPs. **Figure** **4** shows the representative TEM images of the sequential transition of two AuNPs with diameters of 12.4, and 12.9 nm when irradiated at an electron dose rate of 6.6 × 10^4^ e^−^ Å^−^^2^s^−1^. More importantly, two NPs placed at *d_r_* of 1.4 nm show slight misorientation of lattice planes between them. Upon continuous irradiation, the NPs approached each other by reducing the misalignment of lattice planes through a 3D rotation of the particles (Figure 4C,D). This induced the reconstruction of the fused structure by redistributing the atomic density, which also compensated for the misorientation of lattice planes at the neck region to eliminate the twin boundary (Figure 4E,F). Nonetheless, even after 51 min of continuous beam exposure, the coalesced structure was still observed in a dumbbell‐like structure due to the stability of the large‐size AuNPs (Figure 4F). The formation of dumbbell‐shaped structures upon the coalescence of 10 nm AuNPs via five‐fold twin intermediate states is in agreement with previous reports in the literature.^[^ ^19^ ^]^

#### 2.3.1 Neck Growth of Coalesced Structure

The coalescence of the AuNPs initiated a neck‐like structure formation between two closely placed AuNPs without melting the NPs at the edges.^[^ ^18^, ^23^, ^35^, ^42^, ^43^, ^44^, ^45^, ^46^, ^47^ ^]^ The diameter of the neck‐like structure increased with time due to the transfer of atoms at the interface between the two NPs. Figure 3B shows the presence of lattice fringes with *d* = 0.23 nm around the neck region involved in the aligning of lattice planes (the lower NP in Figure 3B is off‐axis). The growth rate of neck radius *r* can quantitatively describe the kinetics of particle coalescence, disclosing the mechanisms involved in the sintering process of two NPs when they are in contact, facilitated by their shared plasmons. Here, we used a power law relation to approximate the kinetics of neck growth for spherical NP coalescence based on the evaluation of the neck diameter *D_n_* as a function of *t*.^[^ ^48^ ^]^

(1) $$D_{n}=Kt^{a}$$

where *K* is a constant depending upon temperature, atomic volume, the average diameter of particles, surface energy, and diffusivity of materials. Exponential *a* refers to the order of the power in the change of neck radius associated with the coalescence mechanism, such as grain boundary diffusion, surface diffusion, and/or lattice diffusion. The value of *a* is predicted by the classical quantum theory in the range of 1/6–1/7, indicating the surface diffusion and grain diffusion mechanisms.^[^ ^22^, ^34^, ^35^, ^47^, ^48^ ^]^

**Figure 5:**
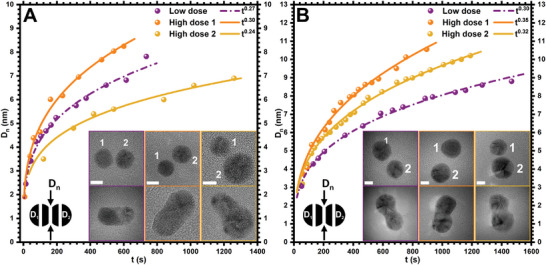
Growth of neck diameter *D_n_* with *t* at different electron dose rates. A) Growth of *D_n_* with *t* for small‐sized AuNPs at a low dose rate (purple, 3.0 × 10^4^ e^−^ Å^−^^2^s^−1^) and high dose rates (orange and gold, 1.2 × 10^5^ e^−^ Å^−^^2^s^−1^). The inset shows TEM images before (top) and after (bottom) neck formation between AuNP pairs with diameters of *D_1_* = 8.1 nm and *D_2_* = 8.3 nm (purple panel), *D_1_* = 7.4 nm and *D_2_* = 8.1 nm (orange panel), and *D_1_* = 6.1 nm and *D_2_* = 9.8 nm (gold panel). B) Growth of *D_n_* with *t* for large‐sized AuNPs at a low dose rate (1.7 × 10^4^ e^−^ Å^−^^2^s^−1^ in purple) and high dose rates (1.4 × 10^5^ e^−^ Å^−^^2^s^−1^ in orange and gold). TEM images before (top) and after (bottom) neck formation between two NPs with diameters of *D*_1_ = 12.6 nm and *D_2_* = 11.6 nm (purple panel), *D_1_* = 12.7 nm and *D_2_* = 12.6 nm (orange panel), and *D_1_* = 10.7 nm and *D_2_* = 14.1 nm (golden panel). The scale bar is 5 nm in all TEM images and graphs show power law *t^a^* fitting in dash‐dotted and solid lines for low and high dose rates, respectively.

We performed the quantitative analyses and the evaluation of *D_n_* with *t* for small and large‐sized AuNPs at two different electron dose rates (**Figure** **5**A,B).

TEM images in the upper rows in the insets of Figure 5 were acquired at *t* = 0 min before the neck formation. The final positions (TEM images in the bottom rows) correspond to the last measurements in the graph after the complete formation of the *D_n_* (see Figures S1–S6 Supporting Information, for sequential TEM frames for small and large‐sized AuNPs). One representative data at a low dose rate (purple) and two (orange and gold) at high dose rates for small and large‐sized AuNPs are presented in Figure 5A and B, respectively. The data was fitted based on the *t^a^* approximation as shown by dash‐dotted and solid lines for low and high dose rates, respectively. The value of *a* at a low dose rate is ≈0.27 and 0.30 for small and large‐sized AuNPs, respectively. However, at a higher dose rate, the value of *a* is 0.30 and 0.35 for small and large‐sized AuNPs, respectively. The variation in the value of *a* given in all observations is due to the difference in the NP diameters and electron dose rates.

It is expected that the neck grows faster not only for large‐sized NPs but also at a higher dose rate (data in orange) due to the accelerated atomic diffusion toward the interface, which persists in all observations (Figure 5). Hence the rate of *D_n_* is low at a low electron dose rate and for smaller NPs. The value of *a* in our data falls within the range of 1/4 to 1/3, indicating surface diffusion as a dominant mass transport mechanism for the coalescence of crystalline particles (Figure 5).^[^ ^6^, ^35^ ^]^ The quantified range of *a*, however, differs from the predictions suggested by classical quantum theory in all observations.^[^ ^34^, ^47^ ^]^ Conversely, previous studies using kinetic Monte Carlo simulations suggest that the coalescence of crystalline particles is impacted by a facet‐mediated surface diffusion, which can enhance the value of *a* from ≈1/7 up to 1/3.^[^ ^22^, ^48^ ^]^ Therefore, the difference in the value of *a* in our case can be justified based on the experimental conditions, such as the electron dose rate, NP size and shape, distance between the NPs before coalescence, and temperature at which NPs are observed.

#### 2.3.2 Growth of Coalesced Structures

**Figure 6:**
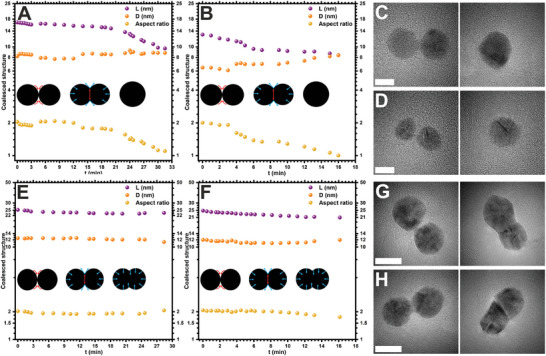
Growth of coalesced structure. A,B) Change in the coalesced structure of small‐sized AuNPs with *t* at electron dose rates of 3 × 10^4^ and 2.5 × 10^5^ e^−^ Å^−^^2^s^−1^, respectively. C,D) The corresponding TEM images of small‐sized AuNPs before (left) and after (right) complete coalescence (scale bar 5 nm). E,F) Change in the coalesced structure of large‐sized AuNPs at electron dose rates of 1.7 × 10^4^ and 1.4 × 10^5^ e^−^ Å^−^^2^s^−1^, respectively. G,H) The corresponding TEM images of large‐sized AuNPs before (left) and after coalescence (right) (scale bar 10 nm). All TEM images in the left column show the initial coalesced structure just after starting the neck formation by assuming *t* = 0. Insets show the schematic diagrams of the coalesced structure growth, where red and blue arrows highlight atom diffusion and surface tension, respectively.

The structural evolution of coalesced NPs after the neck formation and growth was found to be impacted by the size, shape, and crystal structure of NPs. Furthermore, experimental parameters such as sintering time, temperature, electron dose rate, and time of coalesced structure relaxation also play a crucial role in coalescence.^[^ ^49^ ^]^ We monitored the growth of the coalesced structure by measuring the change in the length *L*, the average diameter *D* of two spherical NPs, and the aspect ratio (*L*/*D*) of the structure with time *t* (**Figure** **6**). The small‐sized AuNPs at a dose rate of 3 × 10^4^ e^−^ Å^−^^2^s^−1^ resulted in a coalesced structure (Figure S7; Movie S2, Supporting Information) with 16.8 nm in *L* and an average *D* of 8.2 nm upon neck formation between two NPs (Figure 6A,C, left TEM image). The continued exposure to the electron beam for 32 min reconstructed the coalesced structure into a spherical shape with ≈9.4 nm in *D* (Figure 6C, right TEM image). The surface area and volume of the new spherical structure were ≈278 and 435 nm^3^, respectively. These values were ≈423 nm^2^ for surface area and 578 nm^3^ for volume just after the exposure of an electron beam for spherical NPs (i.e., the sum of both NPs before the coalescence).

However, the decrease in the surface area by 34% is associated with a decrease in the size of the coalesced spherical structure compared to the combined surface area of individual NPs at *t* = 0. The coalescence of small‐sized AuNPs at a low dose rate changed the aspect ratio from 2 to 1.1 (Figure 6A,C). This reflects the decrease in *L* and increase in *D* of the coalesced structure with *t*. Similarly, the coalescence of small‐sized AuNPs at a high dose rate of 2.5 × 10^5^ e^−^ Å^−^^2^s^−1^ (Figure S8; Movie S3, Supporting Information), furnished coalesced structure with 13 nm in *L* and 6.5 nm in *D* (Figure 6B,D, left TEM image). The coalesced structure was transformed into a spherical shape with 8.4 nm in *D* by the reconstruction of the whole structure in 16 min where the aspect ratio was decreased from 2 to 1 (Figure 6D, right TEM image). Here, the surface area and volume of the new spherical coalesced structure were approximated as 222 nm^2^ and 310 nm^3^, respectively. Whereas the total surface area and volume of two spherical NPs at *t* = 0 were ≈281 nm^2^ and 320 nm^3^, respectively. A decrease in the surface area by 21% with almost the same volume was observed for the coalesced structure. The above results suggest that for two closely placed small‐sized AuNPs, the rate of transformation and reconstruction of the coalesced structure is much faster under a higher electron dose rate, indicating surface and grain boundary diffusion mechanisms. This is attributed to the high rate of atomic diffusion across the surface and through the coalesced structure. The atomic diffusion was observed along the longitudinal periphery at the neck region and toward the center for growth of the neck and structural transformation to relax the structure to a spherical shape (insets in Figure 6A,B; Figures S7 and S8, Movies S2 and S3, Supporting Information). The reconstructed structure displayed a decrease in the *L* and aspect ratio and an increase in the *D* of the coalesced structure.

The coalescence of large‐sized AuNPs was slightly different than small‐sized AuNPs when studied at dose rates of 1.7 × 10^4^ and 1.4 × 10^5^ e^−^ Å^−^^2^s^−1^ (Figure 6E–H; Figures S9 and S10, Supporting Information). Unlike small‐sized AuNPs, here, a dumbbell‐like reconstructed structure was formed under a low dose rate (Figure 6G, right TEM image; Figure S9 and Movie S4, Supporting Information). Throughout the growth of the coalesced structure, a 7% decrease in the *L* and a 9% decrease in *D* with nearly the same aspect ratio was observed after 28 min (Figure 6E). The electron dose rate (1.4 × 10^5^ e^−^ Å^−^^2^s^−1^) resulted in a change in the aspect ratio from 2 to 1.7 indicating the decrease in *L* of the coalesced structure from ≈25 to 21 nm in 16 min with nearly the same *D* (12 nm) of the coalesced structure (Figure 6F). The decrease in *L* presents the formation of a rod‐shaped coalesced structure (Figure 6H, right TEM image; Figure S10, Movie S5, Supporting Information), which is different from the coalesced structure given in Figure 6G (right TEM image). The formations of rod‐shaped structures for large coalesced NPs have also been reported in the literature when exposed to electron beam irradiation.^[^ ^6^, ^19^ ^]^ The decrease in the *L* of the coalesced structure demonstrates the growth and transformation of the coalesced structure in large‐sized AuNPs. This is attributed to the diffusion and movement of atoms between two coalesced NPs from the peripheral regions of the structure, where the growth of the structure can be affected by the partial coalescence (insets in Figure 6C,D; Figures S9 and S10, Movies S4 and S5, Supporting Information). Similar observations have been reported previously for platinum and bismuth nanostructures.^[^ ^49^, ^50^ ^]^ The interdiffusion of Au atoms within the structure was noticed with the change in the image contrast which is associated with the reduction of overall surface area and surface energy toward the formation of a stable structure (Figures 3, 4, 5, 6, Figures S7–S10, Movies S1–S5, Supporting Information).^[^ ^6^ ^]^

### 2.4 Repulsion in AuNPs

**Figure 7:**
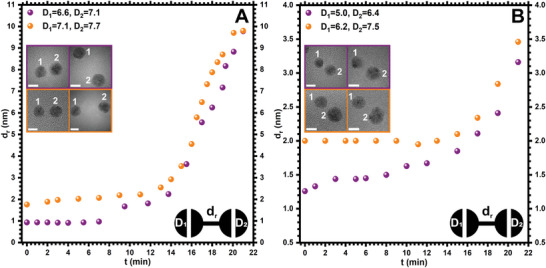
**Repulsion in small‐sized AuNPs under electron beam irradiation**. A) Statistical analyses of the *d_r_* versus *t* for small‐sized AuNPs at an electron dose rate of 3.0 × 10^4^ e^−^ Å^−^^2^s^−1^ (see Movies S6 and S7, Supporting Information). B) Statistical analyses of the *d_r_* versus *t* for small‐sized AuNPs at an electron dose rate of 1.2 × 10^5^ e^−^ Å^−^^2^s^−1^ (see Movies S8 and S9, Supporting Information). Each of the graphs contains two statistical observations (in purple and orange colors) between a pair of AuNPs. The inset shows corresponding TEM images of AuNPs at *t* = 0 and *t* = 21 min. The scale bar for TEM images is 5 nm.

The coalescence of NPs allows for minimizing surface energy for stable structural transformation under the impact of an electron beam. Several factors, such as the *d_r_* between two NPs, size of NPs, beam position, and electron dose rate contribute toward creating either attractive or repulsive forces between pairs of NPs.^[^ ^24^ ^]^ Surprisingly, we found that not all NPs undergo coalescence when exposed to an electron beam. Instead, we found that small and large‐sized AuNPs also undergo repulsion. More importantly, in some instances, we also observed initial attraction followed by repulsion in large‐sized AuNPs. **Figure** **7** and Movies S6–S9 (Supporting Information) show the change in the *d_r_* between pairs of spherical AuNPs as a function of increasing *t* for small‐sized AuNPs at two different electron dose rates.

For example, for a spatially isolated pair of small‐sized AuNPs with *D_1_* and *D_2_* of 6.6 and 7.1 nm, the *d_r_* was 1.0 nm at *t* = 0 (**Figure** **7**A, purple panel; Figure S11 and Movie S6, Supporting Information). Upon irradiation at a dose rate of 3 × 10^4^ e^−^ Å^−^^2^s^−1^ for 21 min the *d*_r_ increased to ≈10 nm. Further exposure up to 29 min, the *d_r_* was increased to ≈18 nm (Figure S11F, Supporting Information). In another set of experiments for two AuNPs with *D_1_* and *D_2_* of 7.1 and 7.5 nm, placed at a *d_r_* of 1.8 nm at *t* = 0 increased to ≈10 nm in 21 min (Figure 7A, orange panel; Figure S12 and Movie S7, Supporting Information). Based on the above observations, the rates of repulsion between pairs of AuNPs can be calculated as 0.4 nm min^−1^. On the other hand, the repulsive behavior at a higher electron dose rate (1.2 × 10^5^ e^−^Å^−^^2^s^−1^) was much lower with ≈0.08 nm min^−1^ (Figure 7B). For example, *d_r_* between two AuNPs with *D_1_* and *D_2_* of 5 and 6.4 nm increased from 1.3 nm at *t* = 0 to 3.2 nm at *t* = 21 min (Figure 7B, purple panel; Figure S13, and Movie S8, Supporting Information). Similarly, for another pair of NPs with *D_1_* and *D_2_* of 6.2 and 7.5 nm, *d_r_* increased from 2 nm at *t* = 0 to 3.5 nm at *t* = 21 min (Figure 7B, orange panel; Figure S14 and Movie S9, Supporting Information). Further exposure up to 27 and 33 min, in both cases the *d_r_* was increased to ≈3.8 and 6.5 nm, respectively (Figures S13F and S14F, Supporting Information).

**Figure 8:**
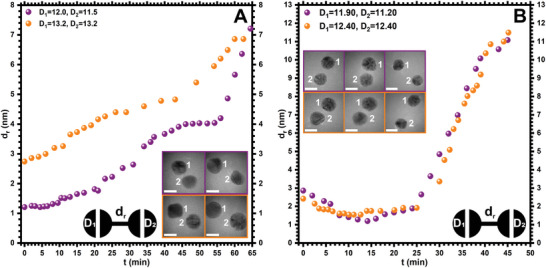
**Repulsion in large‐sized AuNPs under electron beam irradiation**. A) Statistical analyses of the *d_r_* versus *t* for large‐sized AuNPs at an electron dose rate of 1.7 × 10^4^ e^−^Å^−^^2^s^−1^ (see Movies S10 and S11, Supporting Information). B) Statistical analyses of the *d_r_* versus *t* for large‐sized AuNPs at an electron dose rate of 1.4 × 10^5^ e^−^Å^−^^2^s^−1^ (see Movies S12 and S13, Supporting Information). Each of the graphs contains two statistical observations (in purple and orange colors) between a pair of AuNPs. The inset shows corresponding TEM images of AuNPs at *t* = 0 (left panels), *t* = 12 (B, middle panel), *t* = 45 (B, right panel), and *t* = 65 (A, right panel) min. The scale bar for TEM images is 5 nm.

Interestingly, in large‐sized AuNPs, rates of repulsion at a lower electron dose rate of 1.7 × 10^4^ e^−^Å^−^^2^s^−1^ was much lower than that of small‐sized AuNPs (**Figure** **8**A; Figures S15 and S16, Movies S10 and S11, Supporting Information). The observed 0.08 nm min^−1^ rate of repulsion in large‐sized AuNP at a low electron dose rate was similar to that observed at a high electron dose in small‐sized AuNPs. This suggests that the rate of repulsion is also impacted due to increase in the size of NPs. For example, in the case of large‐sized AuNPs, repulsive forces are dominated at a *d_r_* of 1.2 (Figure 8A, purple panel, Figure S15, Movie S10, Supporting Information) and 2.7 nm at a low electron dose rate (Figure 8A, orange panel; Figure S16, Movie S11, Supporting Information).

Surprisingly, at a higher dose rate of 1.4 × 10^5^ e^−^Å^−^^2^s^−1^ the large‐sized AuNPs showed a different behavior (Figure 8B; Figures S17 and S18, Movies S12 and S13, Supporting Information). For example, for two AuNPs with *D_1_* and *D_2_* of 11.9 and 11.2 nm, the *d_r_* of 2.9 nm, the attractive forces are dominated in the first 10–15 min as suggested by a decrease in the *d_r_* to 1.2 nm (Figure 8B, purple panel; Figure S17A–C, Movie S12, Supporting Information). Similarly, for another set of AuNPs, the *d_r_* decreased from 2.4 to 1.6 nm (Figure 8B, orange panel; Figure S18A–C and Movie S13, Supporting Information). However, in the second phase, repulsive forces dominate over the attractive forces and *d_r_* increased in both sets of NPs from 1.2 to 11 nm and 1.6 to 11.5 nm for the next 30 min, respectively (Figure 8, Figures S17D–F, S18D–F, Movies S12 and S13, Supporting Information). The rates of NP diffusions can be approximated as 0.1 and 0.3 nm min^−1^ for attractive and repulsive regimes, respectively, under a higher electron dose rate.

The above observations suggest that the particle size, electron dose rate, *d_r_* and difference in the size of NP pairs affect the interaction behavior under in situ TEM imaging. This is attributed to the charging dynamics, and surface interactions, which lead to varied repulsion behaviors in small and large‐sized AuNPs under electron beam irradiation. The interaction of high‐energy electrons with NPs causes ionization and excitation of the atoms. This may lead to the charging of the NPs due to secondary electron generation.^[^ ^51^, ^52^ ^]^ The charging effect induces the dielectric forces on NPs. In addition to the diffusion, the surface charge such as beam‐induced dipole allows attraction between the spatially isolated NP pairs. However, repulsion is favorable when a multipolar surface is generated. Batson et al. demonstrated that polarization can be induced by passing the electron beam in the vicinity of two isolated NP pairs.^[^ ^24^ ^]^ It has been observed that the passing of a swift electron beam can produce 2–20 pN instantaneous transverse force. Such force creates multipolar surface  on each closely placed NPs driving the NPs to repel from each other.^[^ ^24^, ^37^ ^]^ The induced dipole moment can lead to the creation of an electric field around each NP. The interaction of electron beam between charged NPs, induced multipoles, and electric field around polarized NPs leads to the generation of electrostatic forces, and, consequently, strong repulsion between the NPs.^[^ ^11^ ^]^

We have observed that there is a significant difference in the rate of diffusions for repulsive behavior in small‐sized AuNPs for the same *t* in all four experiments under low and high electron dose rates. This dynamic behavior can be explained based on the interaction between the electron beam and the NPs, which is relatively gentle under the low electron dose rate. Consequently, NPs may have more time for effective charging and polarization. Conversely, in small‐sized AuNPs at a higher electron dose rate, rapid charging and polarization may result in NPs without enough time for effective polarization, and redistribution of charges over the surface. As a result, the interaction between the induced multipoles and the resulting electric field gets weaker, causing the NPs to repel each other at a shorter distance. Additionally, the ineffective polarization may compete with attractive forces, which are more prominent in the case of large‐sized AuNPs under a higher electron dose rate, thereby reducing the effects of repulsive forces.

On the other hand, the rate of repulsion for large‐sized AuNPs is low compared to that of small‐sized AuNPs. This is attributed to charging dynamics, surface effects, and size‐dependent electron interaction. The interaction between electron beam and large‐sized AuNPs at low electron dose rate can accommodate and redistribute more charges on their surface due to higher volume without approaching the high charge densities, reducing the charge accumulation, and mitigating the probability of strong electrostatic repulsion. Furthermore, the lower surface curvature of large‐sized NPs also reduces the polarization and charge accumulation on the surface which contrasts with small‐sized NPs. Nonetheless, a higher electron dose rate can be manipulated with large‐sized AuNPs in two regimes, attractive and repulsive with *t*. A higher electron dose rate creates rapid and substantial charging on large‐sized AuNPs, which may create attractive forces between a pair of AuNPs due to induced multipoles and unlike charges on NPs. The continuous exposure of a higher electron beam induces more charges on the surface. Because of more surface defects, lattice mismatching, and higher charge density with limited relaxation time for charge redistribution, repulsive forces are dominated in the second phase. Consequently, stronger repulsive forces are formed under a high electron dose rate than a low electron dose rate in a shorter electron beam exposure time. Similar behavior has also been observed previously in silver nanocrystals and iron oxide NPs, which are attributed to alike charge repulsion due to lattice mismatching.^[^ ^17^, ^53^ ^]^ However, in our case, the lattice mismatching was also observed during coalescence indicating the contribution of more complicated electrostatic interactions in NPs' repulsion (Figure 4).

Based on the given data in Figures 7 and 8 and additional observations, it was found that the minimum thresholds to achieve repulsive forces between a pair of small‐sized AuNPs are approximately *d_r_* = 1.0–1.5 nm and 1.3–2.0 nm for low and high electron dose rates, respectively. Importantly, in our experiments, it also depends on the crystal structure and size of each NP. Similarly, the thresholds of *d_r_* in the case of large‐sized AuNPs for repulsive characters were between 1.2–3.0 and 2.0–3.0 nm at low and high dose rates, respectively. Generally, the minimum threshold of *d_r_* for repulsive behavior increased with the increase of NP size and electron dose rate due to surface charge dynamics. Depending on the size and structure of the NPs, in some cases, it was also observed that a pair of NPs approached coalescence beyond (3.4 nm) the threshold distance (Figure S19 and Movies S14 and S15, Supporting Information). On the other hand, NPs may also develop repulsive forces before the threshold distance which may be justified based on a detailed investigation of the understanding of size‐depended electronic structure, localized surface features of NPs, and their interactions with a beam of electron. During the investigation of the repulsive character of AuNPs, dynamic behaviors such as rotation, structural transformation, and perturbation of NPs were observed. Such behaviors are usually observed upon the interaction of electron beams with NPs and may also be considered as a mechanistic approach in repulsive behavior.^[^ ^15^, ^23^, ^25^ ^]^

## 3 Conclusion

Metal NPs are sensitive to a high‐energy electron beam, which results in their structural transition or dimensional change. However, by careful manipulation of the electron beam dose rate and the properties of metal NPs, new morphological features and optoelectronic properties could be achieved. Studies on the structural transition of AuNPs under a wide range of conditions are well documented in the literature. In recent years, an extensive body of experimental and computational research has been done to understand the dynamics of the coalescence of AuNPs. The experimental and theoretical understanding of the coalescence of a wide range of spherical particles is well established. However, there is a need to investigate other dynamic processes, such as repulsion, attraction, and a combination of attraction and repulsion. We have performed systematic studies to investigate the effect of electron dose rate, NP size, *d_r_*, and the difference in size between two closely placed AuNPs on their dynamics using in situ TEM imaging. More importantly, our results suggest that, in addition to coalescence, NP pairs undergo repulsion or sequential attraction‐repulsion depending on the particle dimension and electron beam dose rate. This finding suggests that electron dose can be utilized not only to create new structures but also to precisely manipulate individual NPs and interparticle distances. Precise manipulation and placement of NPs pave the way for potential applications in photonic and plasmonic nanodevice fabrication. Our experimental results suggest that conventional electron beam dose allows more than one nanoscale dynamics. Here, we have shown the effect of NP size, the *d_r_*, difference in the particle sizes with mutual contact, and electron dose rates on carbon substrates. The results encourage further investigation of the NPs with different substrates and surface functionalities, surface charges, and morphologies. Such experimental approaches, coupled with computational simulation, will provide crucial insights into the mechanistic details of largely unexplored nanoscale dynamics.

## 4 Experimental Section

### General Material

The aqueous dispersions of AuNPs stabilized in citrate buffer, were obtained from Sigma Aldrich. The AuNPs were used without further purification. Two types of AuNPs with average sizes of 5 nm (conc. 5.5 × 10^13^ particles mL^−1^) and 10 nm (conc. 6.0 × 10^12^ particles mL^−1^) referred to as small and large‐sized AuNPs, respectively, with optical densities of 1 were used. For TEM imaging, 200 mesh copper grids with carbon support film of thickness 28–30 nm were acquired from Agar Scientific.

### Sample Preparation

The original solutions of AuNPs were diluted with water in 1:1 (v/v) and dispersed using ultrasonication for 30 min before specimen preparation for TEM studies. The typical procedure for the specimen preparation was adopted by drop casting the 2 µL of diluted solution on carbon film supported with a copper grid using a micropipette. The excess solvent was removed by blotting with filter paper, and the specimen was dried overnight in the dark.

### Transmission Electron Microscopy (TEM) Imaging

In situ dynamic analysis was performed at 200 kV using Jeol JEM‐F200 S/TEM equipped with Jeol dual energy dispersive X‐ray spectroscopy (EDS) and Gatan OneView camera system. The standard single‐tilt TEM holder from Jeol was used for experimental studies at room temperature. The probe current was controlled by the spot size and condenser lens aperture. The images with 2k resolution and screen movies with 5fps were acquired with an exposure time of 0.01 s for most of the experiments using Gatan DigitalMicrograph (DM) and FastStone Capture software, respectively. The data was analyzed using DM and ImageJ software.

### Size Distribution Analysis of NPs

The size distribution of AuNPs in Figure 1 was analyzed using the ImageJ software by assuming the spherical shape of NPs. However, the irregular shape and fused AuNPs were ignored during the analysis. Before analyzing the NPs, the contrasts of the TEM images were enhanced and normalized by 0.35% saturated pixels followed by the noise reduction using the median filter with a radius of 2 pixels. A threshold was applied for analyzing the size distribution based on the area of spherical NPs. The average sizes of NPs were calculated based on the uniform geometrical normalized distribution (0–1) by analyzing the 1012 and 1309 NPs with a bin size of 0.2 nm for small and large‐sized AuNPs, respectively.

### Statistical Analysis

TEM images and data were pre‐processed to ensure accuracy and clarity. Initially, contrast enhancement and normalization were applied to the original TEM images, followed by noise reduction using median or mean filters to improve the signal‐to‐noise ratio using ImageJ software. The size distribution of the AuNPs was analyzed using ImageJ software, and the average size, along with the standard deviation, is presented. Structural transformations of the AuNPs were imaged and examined, with observations conducted on an average of 25 and 100 observations, respectively, at various time intervals under electron beam irradiation. The results and discussion on the coalescence of NPs were based on an average of 50 observations, with data visualization and processing performed using ImageJ and DM software. As detailed in Sections 2.3 and 2.4, the statistical analyses included spherical shape approximation by drawing circular shapes around the NPs and measuring interparticle distances and diameters using DM software. The data and TEM images presented in Figure 5 (Section 2.3.1) were recorded from six experiments, while those in Figure 6 (Section 2.3.2) were acquired from four experiments. A total of 50 observations were used to analyze neck diameters and the growth of coalesced structures. Repulsion between AuNPs was observed in an average of 60 experiments, with eight representative cases shown in Figures 7 and 8 for small and large‐sized AuNPs. The interparticle distances associated with repulsion were measured using DM software. Twin boundaries and lattice spacings were also identified and measured using DM software. FFT and inverse FFT images were generated from the original TEM images using DM software. Results were consistent and reproducible across all observations.

## Conflict of Interest

The authors declare no conflicts of interest.

## Author Contributions

A.Z., N.N., and M.V. conceived the idea. A.Z. carried out the characterization, TEM imaging, image analysis, and interpretation of the data. M.H. assisted in TEM imaging. A.Z. and N.N. wrote the first draft of the manuscript. All authors contributed during the preparation of the final manuscript.

## Supporting information



Supporting Information

Supplemental Movie 1

Supplemental Movie 2

Supplemental Movie 3

Supplemental Movie 4

Supplemental Movie 5

Supplemental Movie 6

Supplemental Movie 7

Supplemental Movie 8

Supplemental Movie 9

Supplemental Movie 10

Supplemental Movie 11

Supplemental Movie 12

Supplemental Movie 13

Supplemental Movie 14

Supplemental Movie 15

## Data Availability

The data that support the findings of this study are available in the supplementary material of this article.
