# Supplementary material for: In Situ TEM Imaging Reveals the Dynamic Interplay Between Attraction, Repulsion and Sequential Attraction‐Repulsion in Gold Nanoparticles
Source: Small. 2024 Oct 8;20(51):2406943. doi: 10.1002/smll.202406943 (PMC11656691; doi:10.1002/smll.202406943)
Supplement: Supplementary file 1 — Supporting Information [file SMLL-20-2406943-s013.pdf]

NANO · MICRO
small

Supporting Information

for *Small*, DOI 10.1002/smll.202406943

In Situ TEM Imaging Reveals the Dynamic Interplay Between Attraction, Repulsion and Sequential Attraction-Repulsion in Gold Nanoparticles

Abid Zulfiqar, Mari Honkanen, Nonappa and Minnamari Vippola**

Supplementary Information

In Situ TEM Imaging Reveals the Dynamic Interplay Between Attraction, Repulsion and Sequential Attraction-Repulsion in Gold Nanoparticles

Abid Zulfiqar, Mari Honkanen, Nonappa, Minnamari Vippola**

A. Zulfiqar, M. Honkanen, Nonappa, M. Vippola
Faculty of Engineering and Natural Sciences, Tampere University, FI-33720 Tampere, Finland
E-mail: nonappa@tuni.fi, minnamari.vippola@tuni.fi

M. Honkanen, M. Vippola
Tampere Microscopy Center, Tampere University, FI-33720 Tampere, Finland

1. Supplementary Methods

1.1. Calculation of the electron dose rate

In order to determine the electron dose rate (D_r), the probe current (I_p) was measured using the Faraday cup installed in the column connected to an external pico amperemeter with approximately the same values provided by the microscope manufacturer (Jeol, Japan). The electron dose D_e in e^- Å^{-2} was then calculated using the following equation:

$$D_e = \frac{I_p t}{eA}$$

with exposure time t, elementary charge e, and area exposed to the electron beam A.[1]
Where the D_r in e^- Å^{-2}s^{-1} was determined per second by using the D_e:

$$D_r = {}^{D_e}/_{t}$$

1.2. Determining the misorientation of lattice planes

To investigate the coalescence of nanoparticles (NPs) induced by the misorientation of lattice planes, we quantified the misorientation angles at the interface of two NPs using Transmission Electron Microscopy (TEM) images given in Figure 4B. The TEM images were imported to ImageJ, where the contrast of the images was improved to ensure the clear visibility of lattice planes of the NPs. Using the line tool in ImageJ, lattice planes of both NPs were carefully plotted to accurately reflect their orientations. The angle tool in ImageJ was subsequently used to measure the misorientation angle between the plotted lattice planes by selecting the start point of the lattice plane line of the first nanoparticle (NP), the intersection point, and the endpoint of the lattice plane line of the second NP. This process was repeated for multiple lattice planes to obtain statistically significant data. The recorded misorientation angles were then analyzed to understand their influence on the coalescence behavior of the NPs.

2. Supplementary figures

The time on the right corner of each TEM sequential frame shows the total electron beam exposure time *t*.

2.1. Neck growth of coalesced structure

See section 2.3.1. in the main text.

2.1.1. Small-sized gold (Au) nanoparticles (NPs)

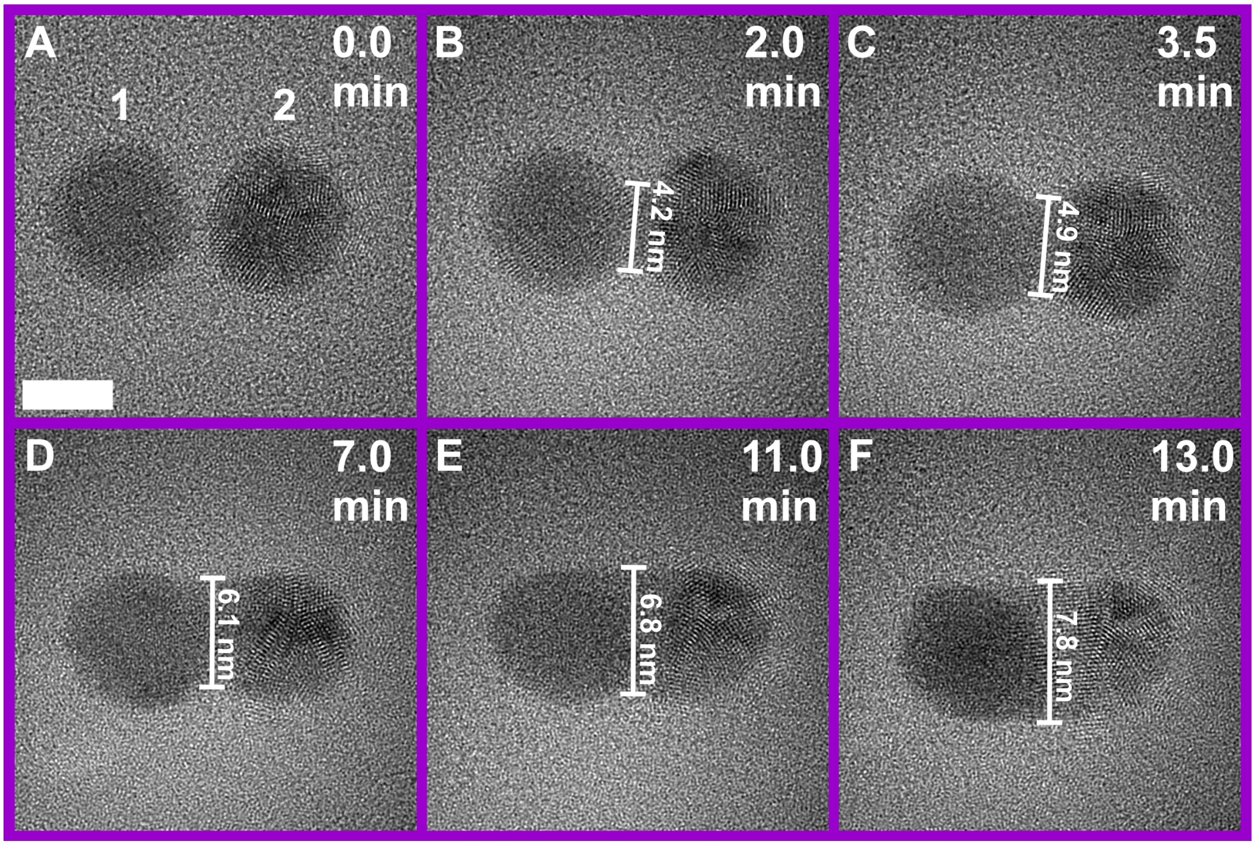

Figure S1. Growth of neck diameter D_n with time t under electron beam exposure in small-sized gold (Au) nanoparticles (NPs) with diameters D_1 and D_2 of 8.1 nm and 8.3 nm at 3.0×10^4 $e^-/Å^2s$ dose rate. The scale bar is 5 nm.

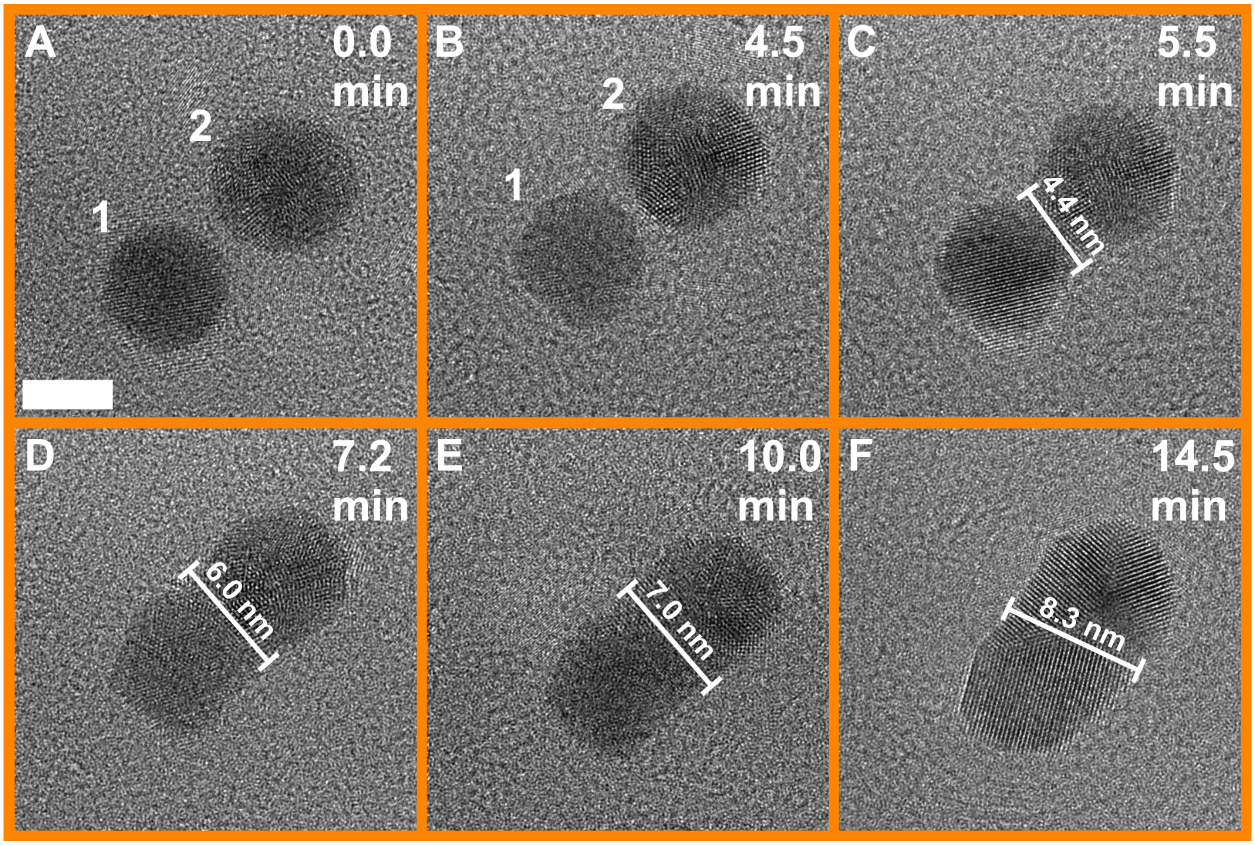

Figure S2. Growth of D_n with t under electron beam exposure in small-sized AuNPs with D_1 and D_2 of 7.4 nm and 8.1 nm at 1.2×10^5 $e^-/Å^2s$ dose rate. The scale bar is 5 nm.

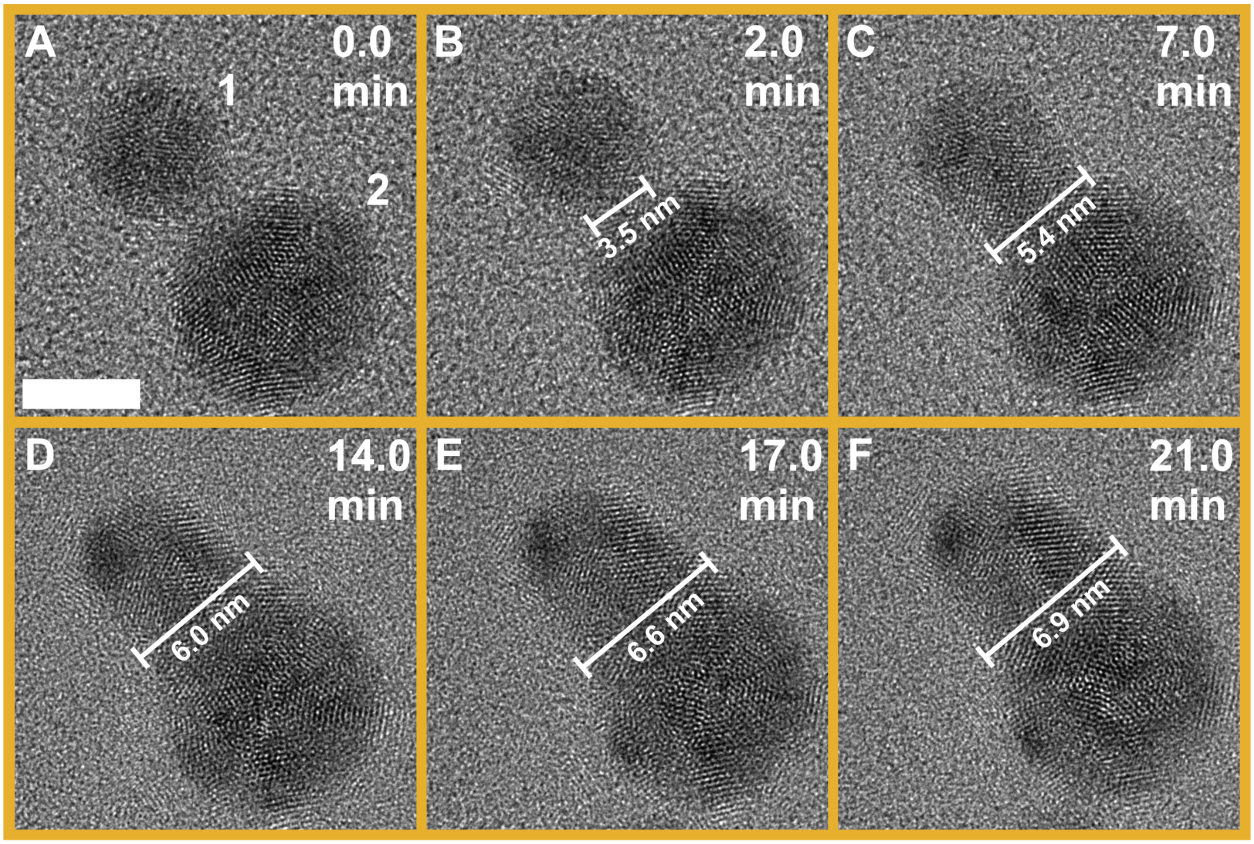

Figure S3. Growth of D_n with t under electron beam exposure in small-sized AuNPs with D_1 and D_2 of 6.1 nm and 9.8 nm at 1.2 × 10^5 $e^-/Å^2s$ dose rate. The scale bar is 5 nm.

2.1.2. Large-sized AuNPs

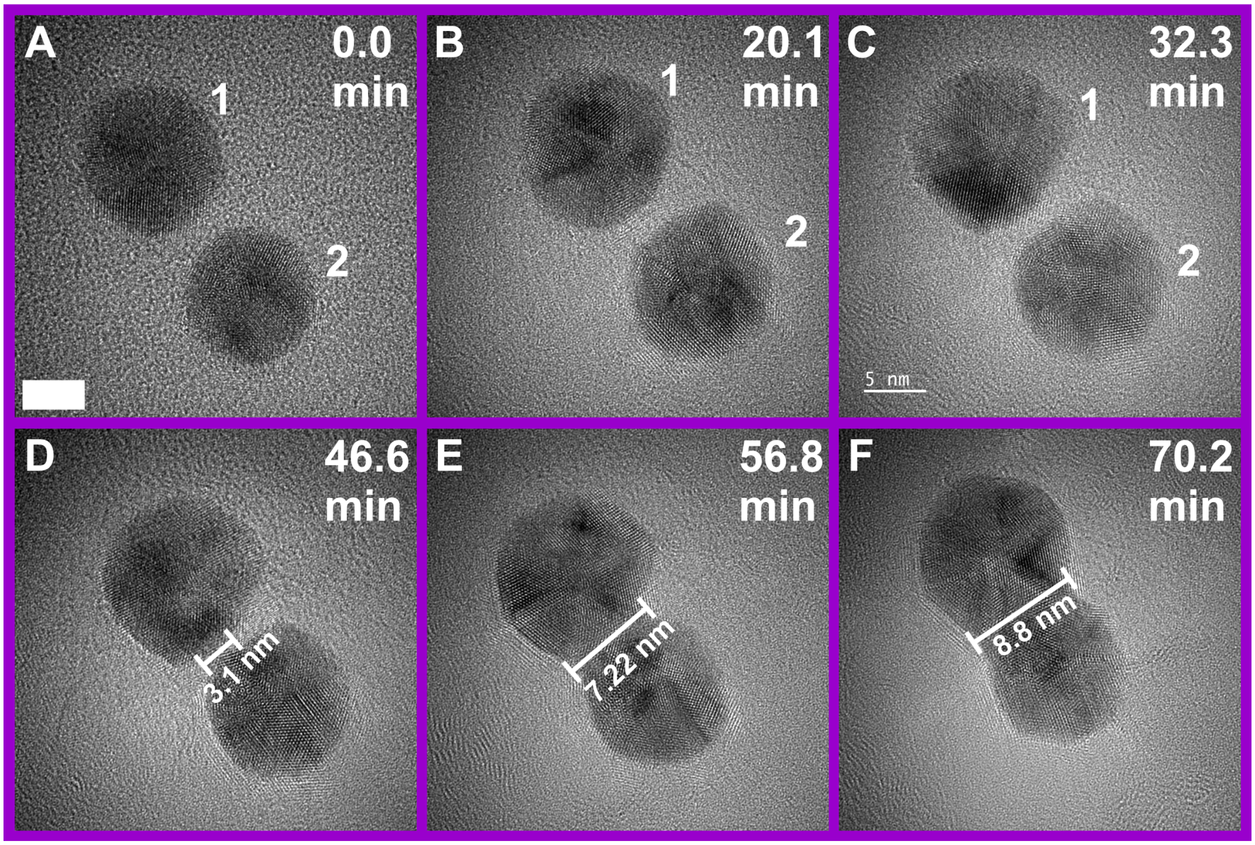

Figure S4. Growth of D_n with t under electron beam exposure in large-sized AuNPs with D_1 and D_2 of 12.6 nm and 11.6 nm at 1.7 × 10^4 $e^-/Å^2s$ dose rate. The scale bar is 5 nm.

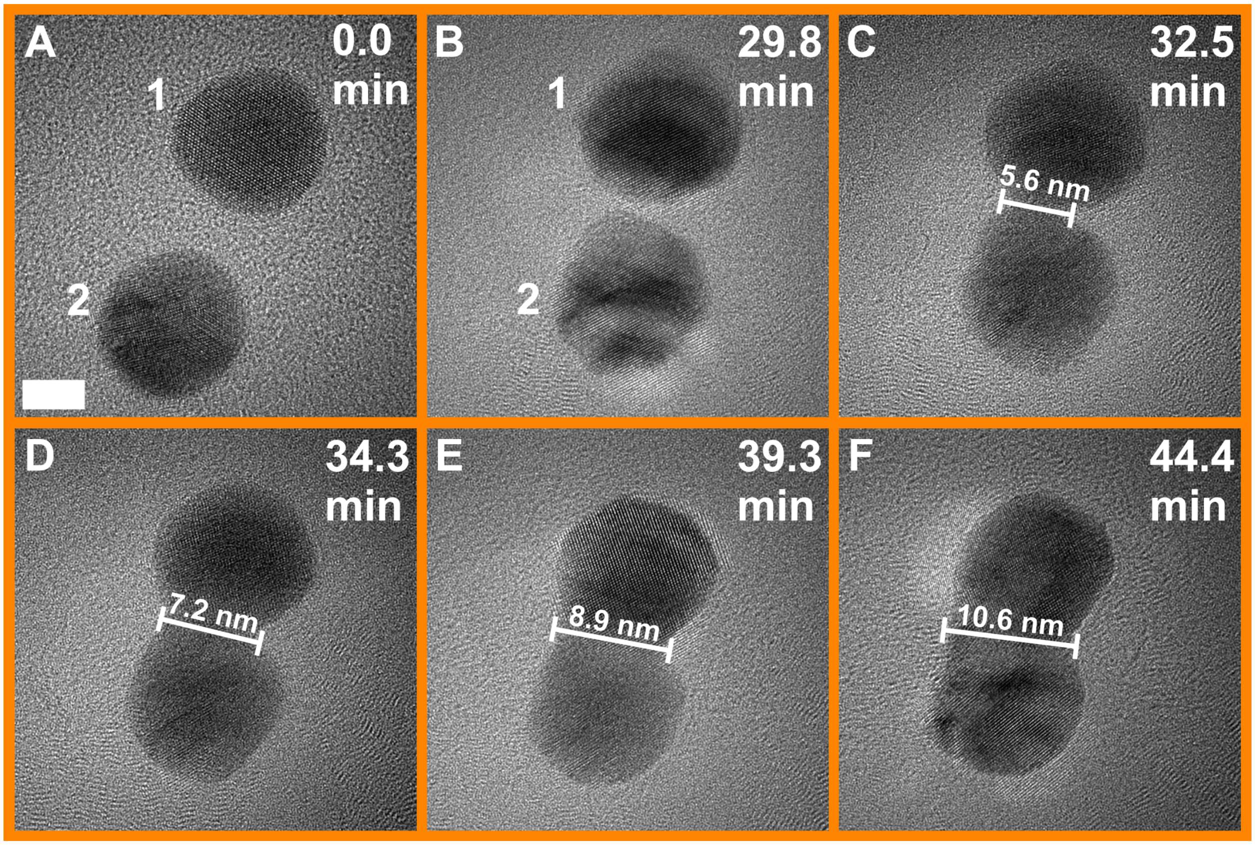

Figure S5. Growth of D_n with t under electron beam exposure in large-sized AuNPs with D_1 and D_2 of 12.7 nm and 12.6 nm at 1.4×10^5 $e^-/Å^2s$ dose rate. The scale bar is 5 nm.

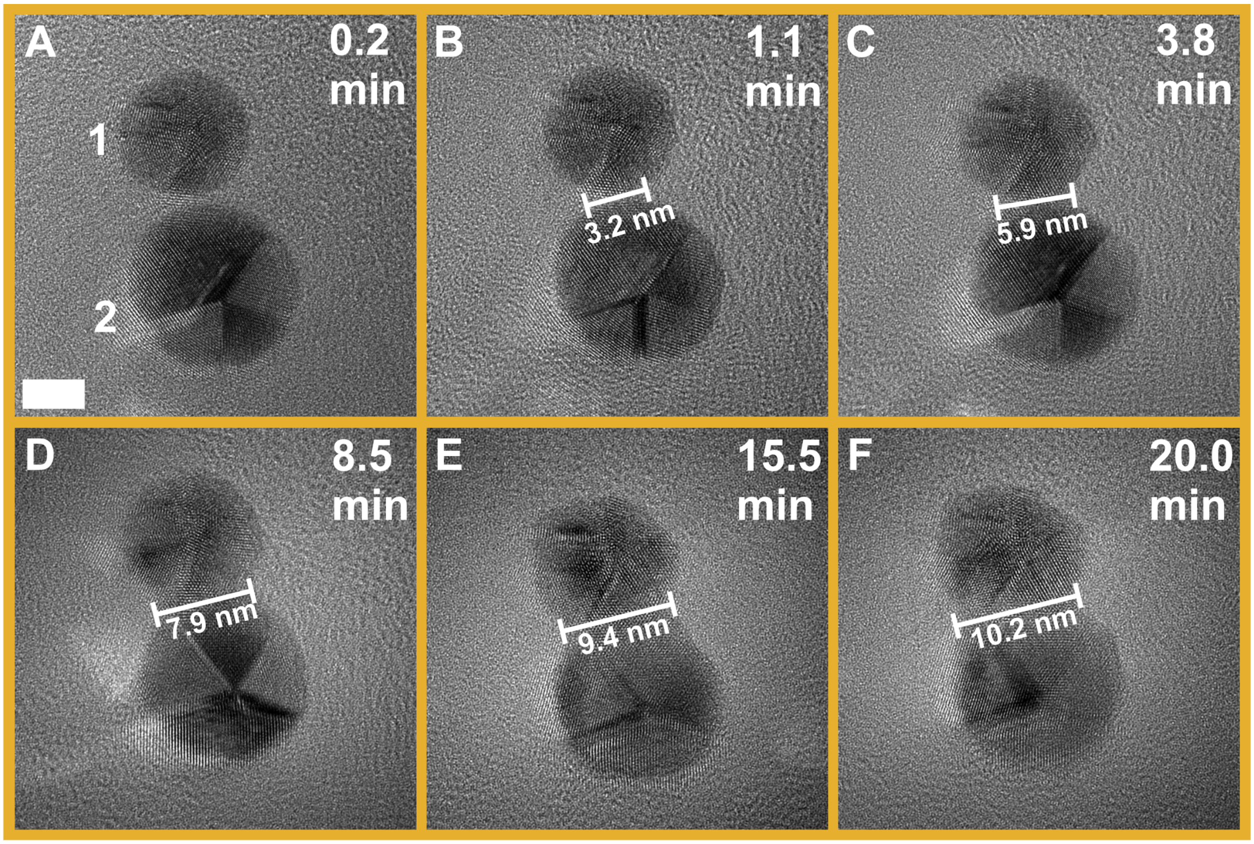

Figure S6. Growth of D_n with t under electron beam exposure in large-sized AuNPs with D_1 and D_2 of 10.7 nm and 14.1 nm at 1.4×10^5 e^-/Å^{2}s dose rate. The scale bar is 5 nm.

2.2. Growth of coalesced structure

See section 2.3.2. in the main text.

2.2.1. Small-sized AuNPs

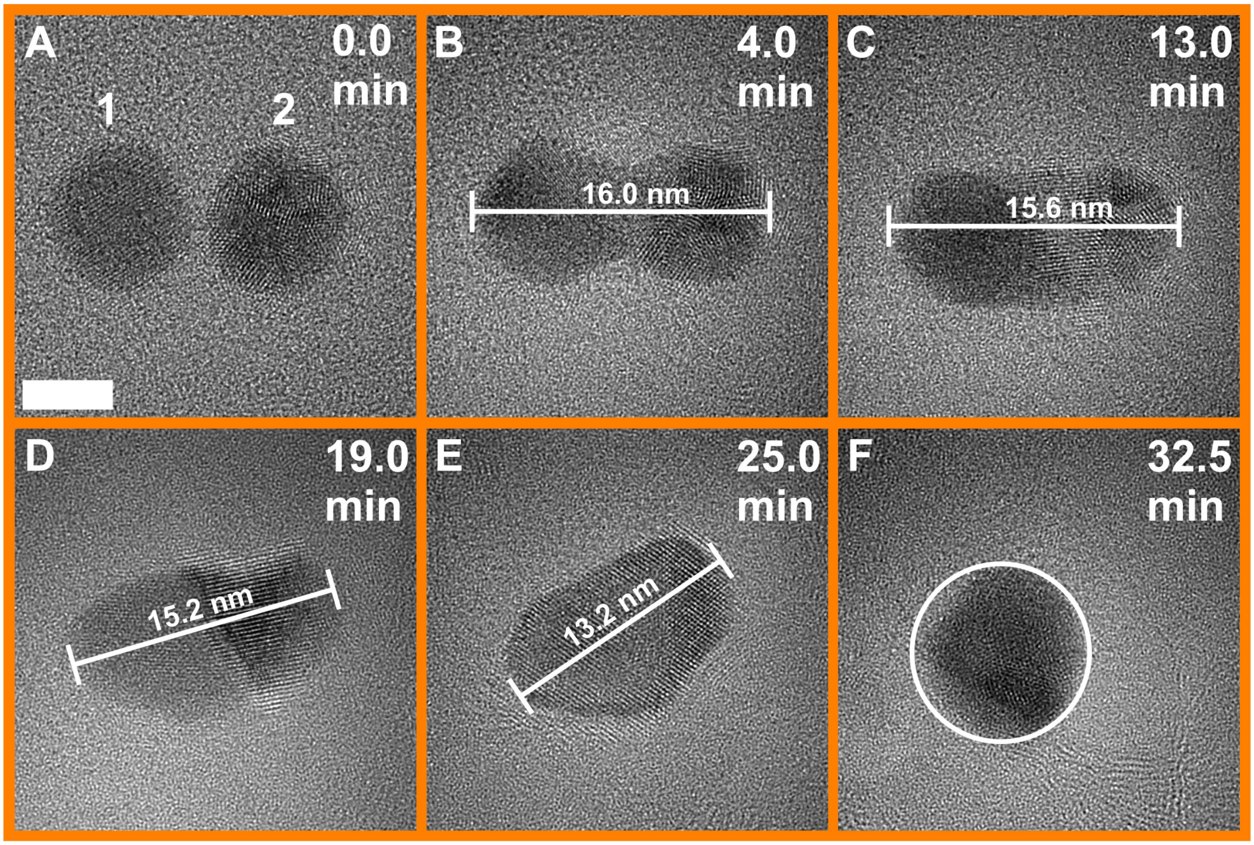

Figure S7. Growth of coalesced structure with t under electron beam exposure in small-sized AuNPs with D_1 and D_2 of 7.4 nm and 8.1 nm at 3×10^4 $e^-/Å^2s$ dose rate. The scale bar is 5 nm.

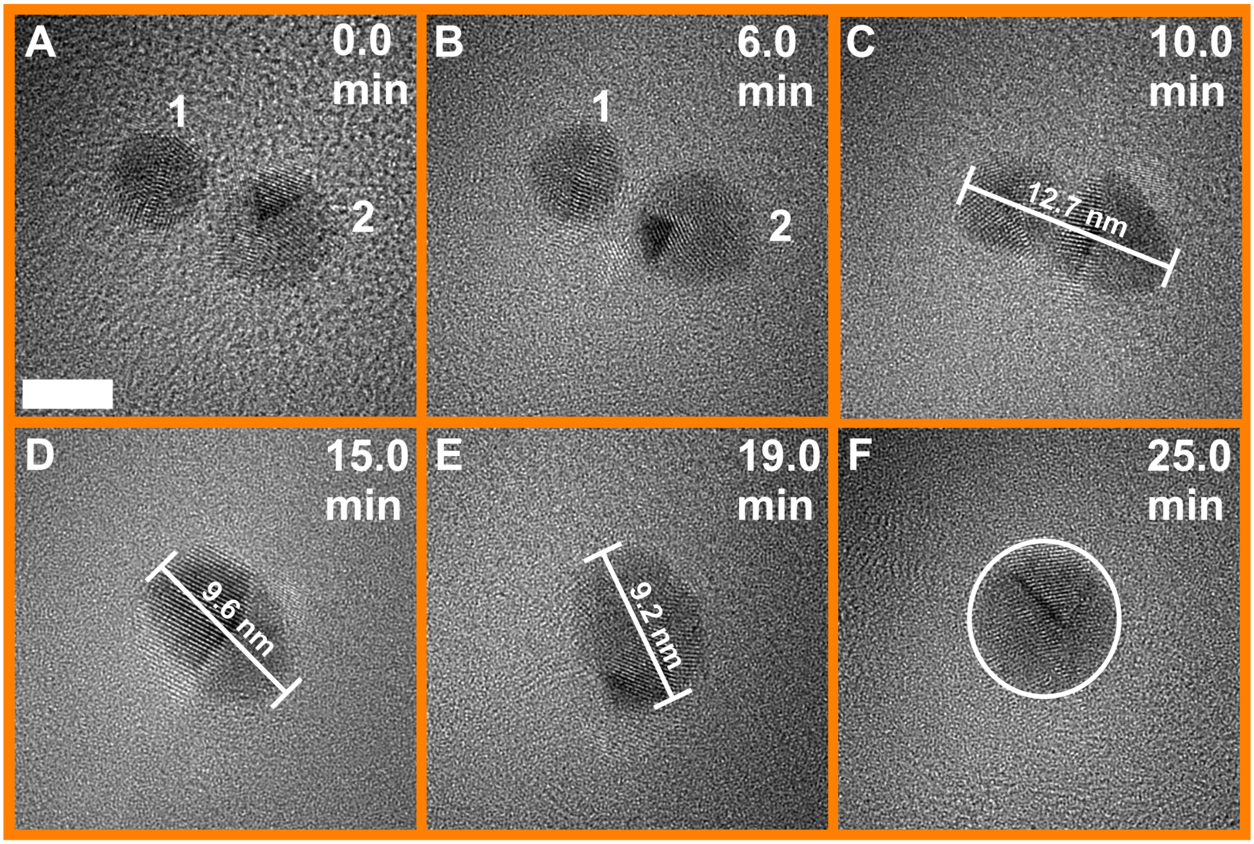

Figure S8. Growth of coalesced structure with t under electron beam exposure in small-sized AuNPs with D_1 and D_2 of 5.8 nm and 7.1 nm at 2.5×10^5 $e^-/Å^2s$ dose rate. The scale bar is 5 nm.

2.2.2. Large-sized AuNPs

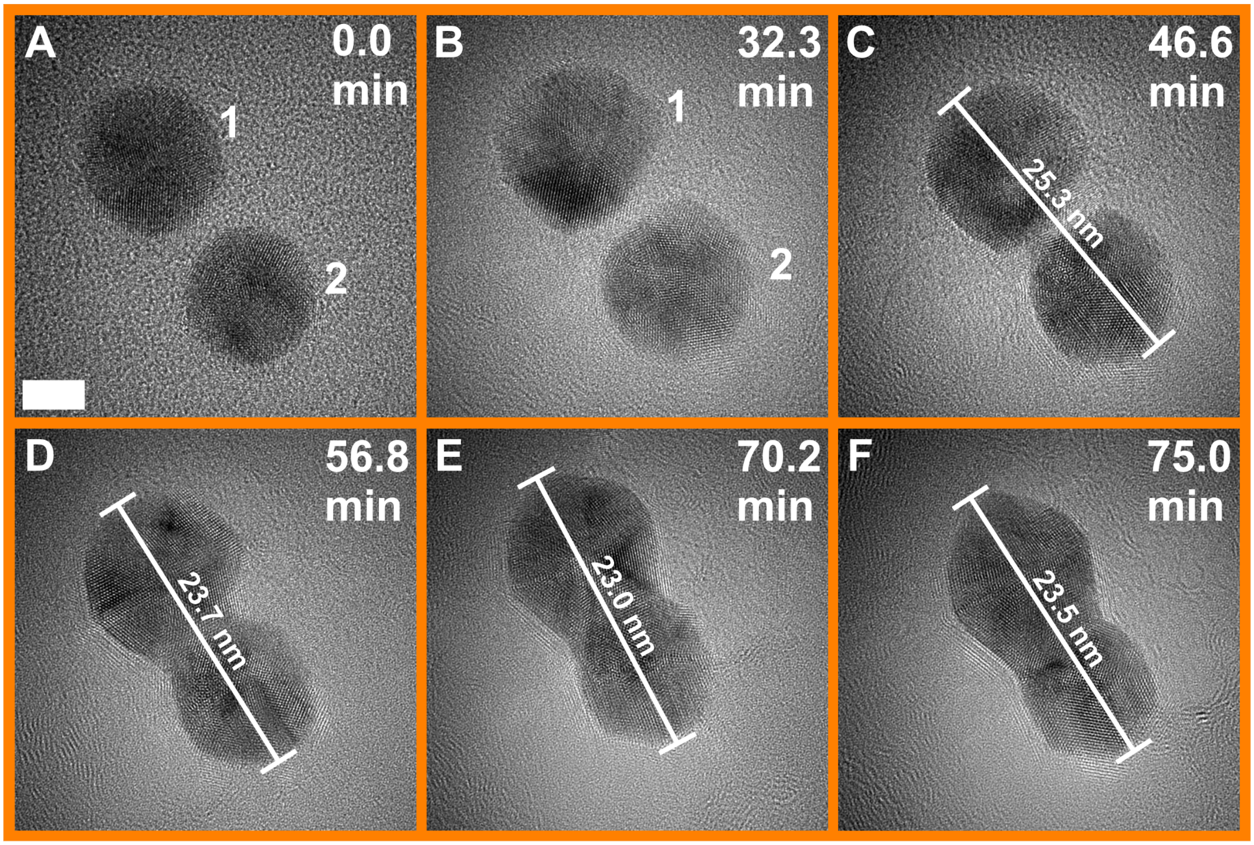

Figure S9. Growth of coalesced structure with t under electron beam exposure in large-sized AuNPs with D_1 and D_2 of 12.6 nm and 11.6 nm at 1.7×10^4 $e^-/Å^2s$ dose rate. The scale bar is 5 nm.

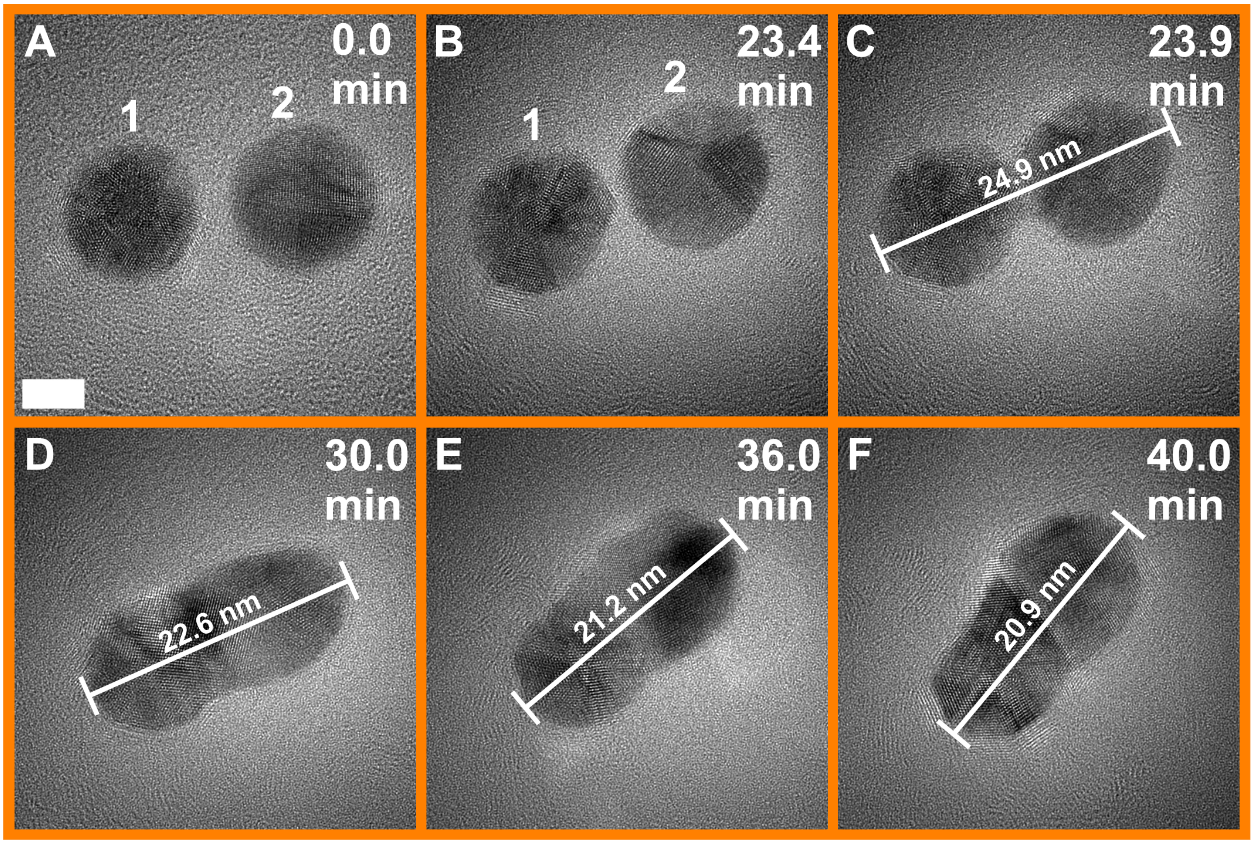

Figure S10. Growth of coalesced structure with *t* under electron beam exposure in large-sized AuNPs with D_1 and D_2 of 11.6 nm and 12.4 nm at 1.4×10^5 $e^-/Å^2s$ dose rate. The scale bar is 5 nm.

2.3. Repulsion of AuNPs

Section 2.4. in the main text.

2.3.1. Small-sized AuNPs

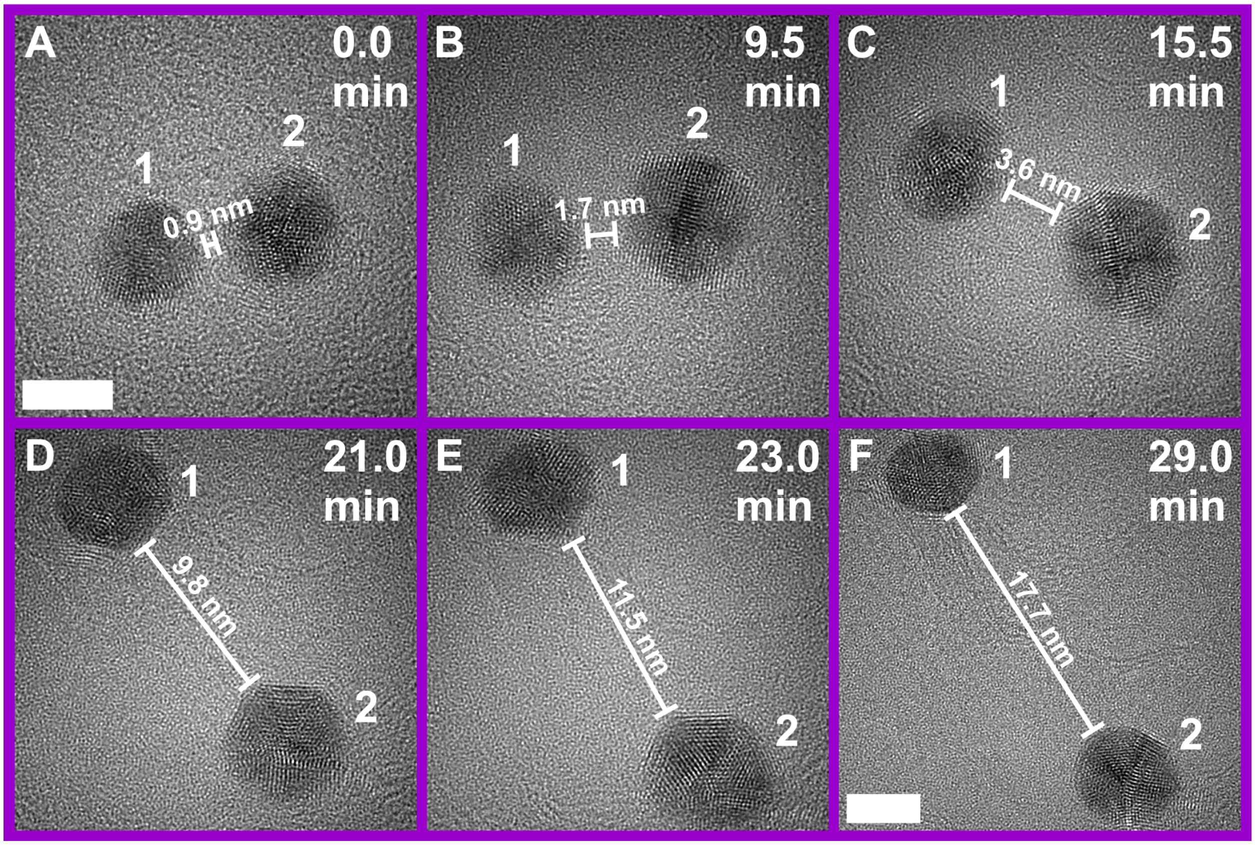

Figure S11. Repulsive character between a pair of small-sized AuNPs with *t* under electron beam exposure with D_1 and D_2 of 6.6 nm and 7.1 nm at 3.0×10^4 $e^-/Å^2s$ dose rate. The scale bar is 5 nm.

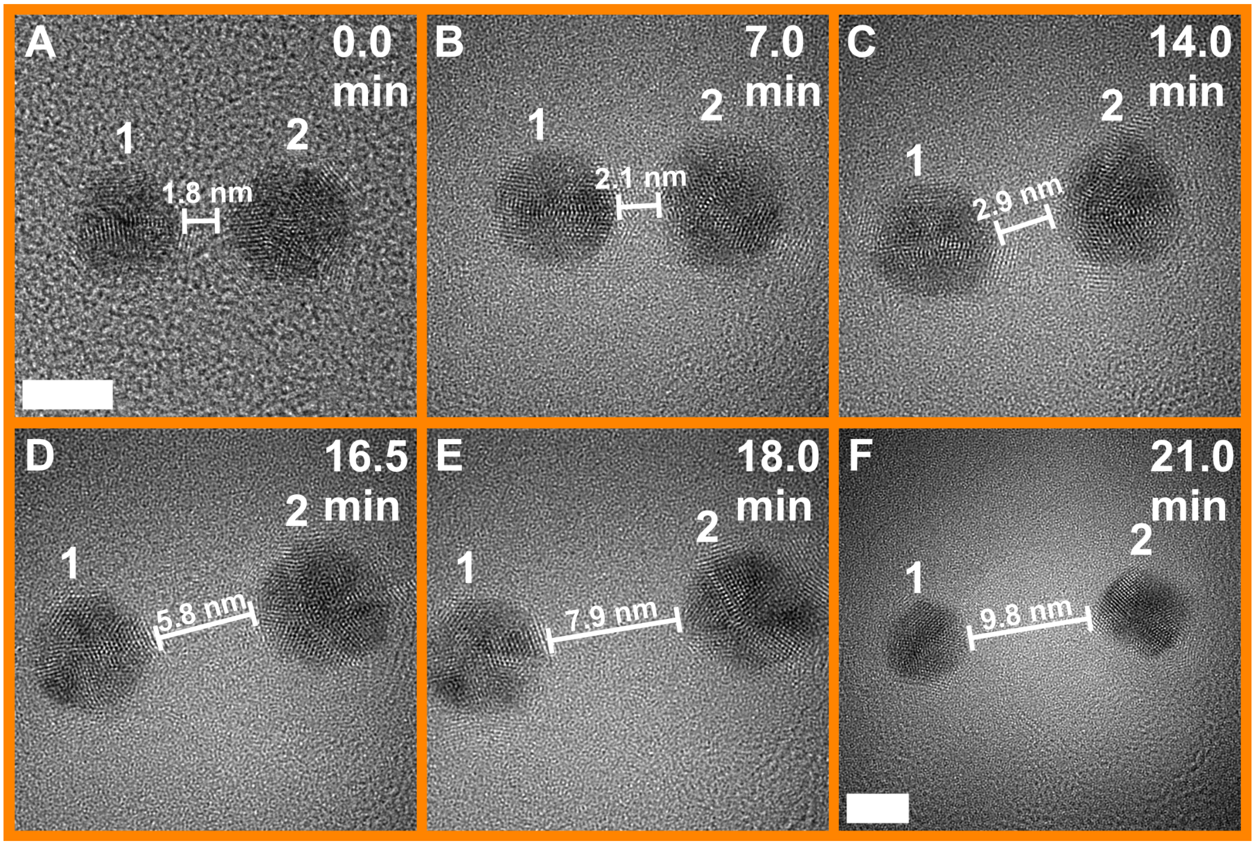

Figure S12. Repulsive character between a pair of small-sized AuNPs with t under electron beam exposure with D_1 and D_2 of 7.1 nm and 7.5 nm at 3.0×10^4 $e^-/Å^2s$ dose rate. The scale bar is 5 nm.

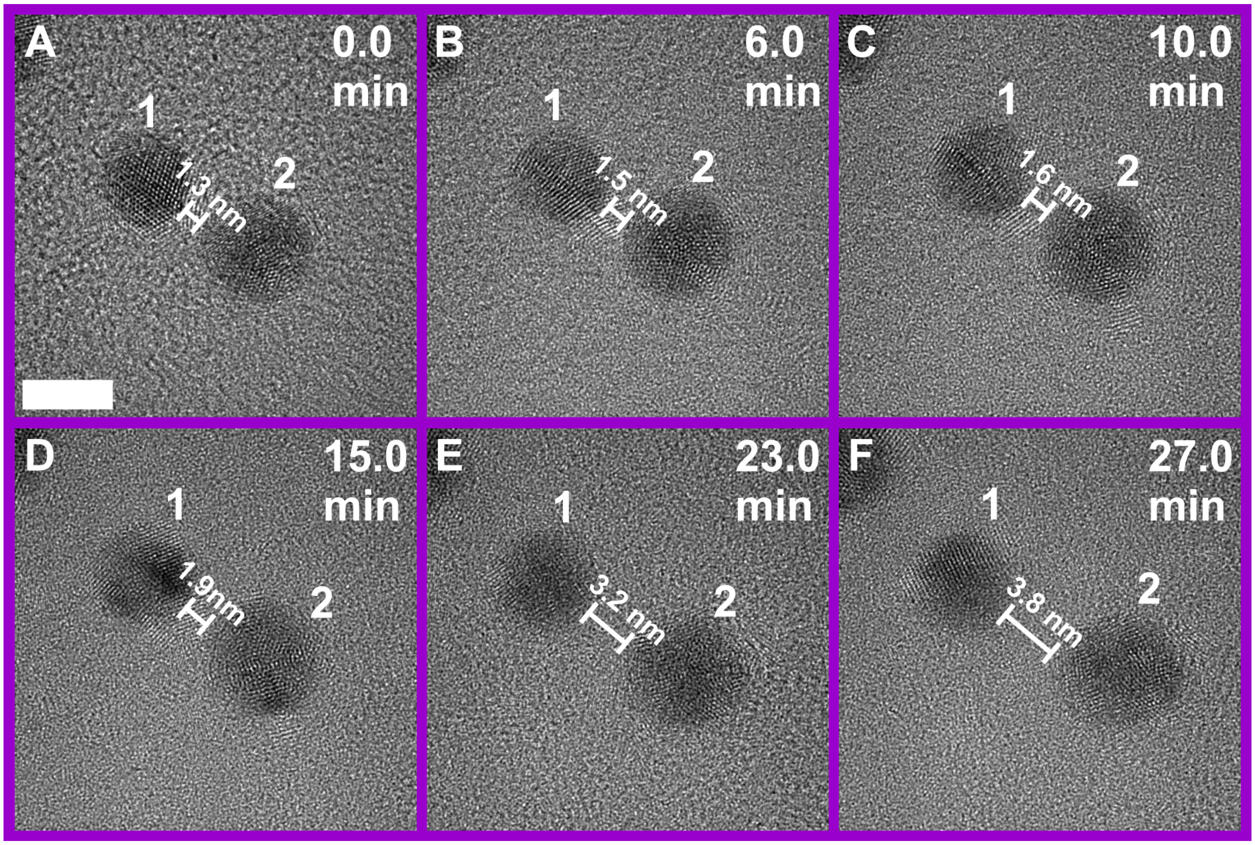

Figure S13. Repulsive character between a pair of small-sized AuNPs with *t* under electron beam exposure with D_1 and D_2 of 5.0 nm and 6.4 nm at 1.2×10^5 $e^-/Å^2s$ dose rate. The scale bar is 5 nm.

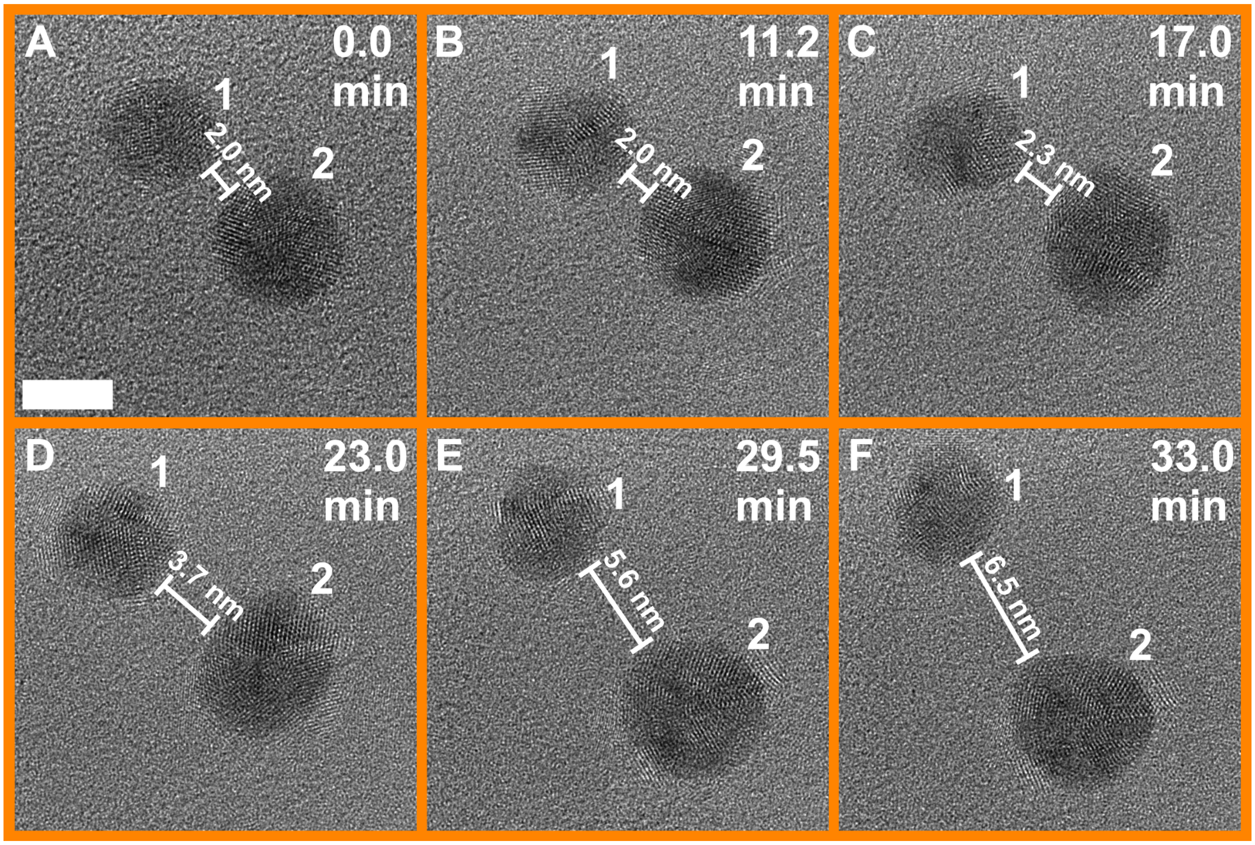

Figure S14. Repulsive character between a pair of small-sized AuNPs with *t* under electron beam exposure with D_1 and D_2 of 6.2 nm and 7.5 nm at 1.2×10^5 $e^-/Å^2s$ dose rate. The scale bar is 5 nm.

2.3.2. Large-sized AuNPs

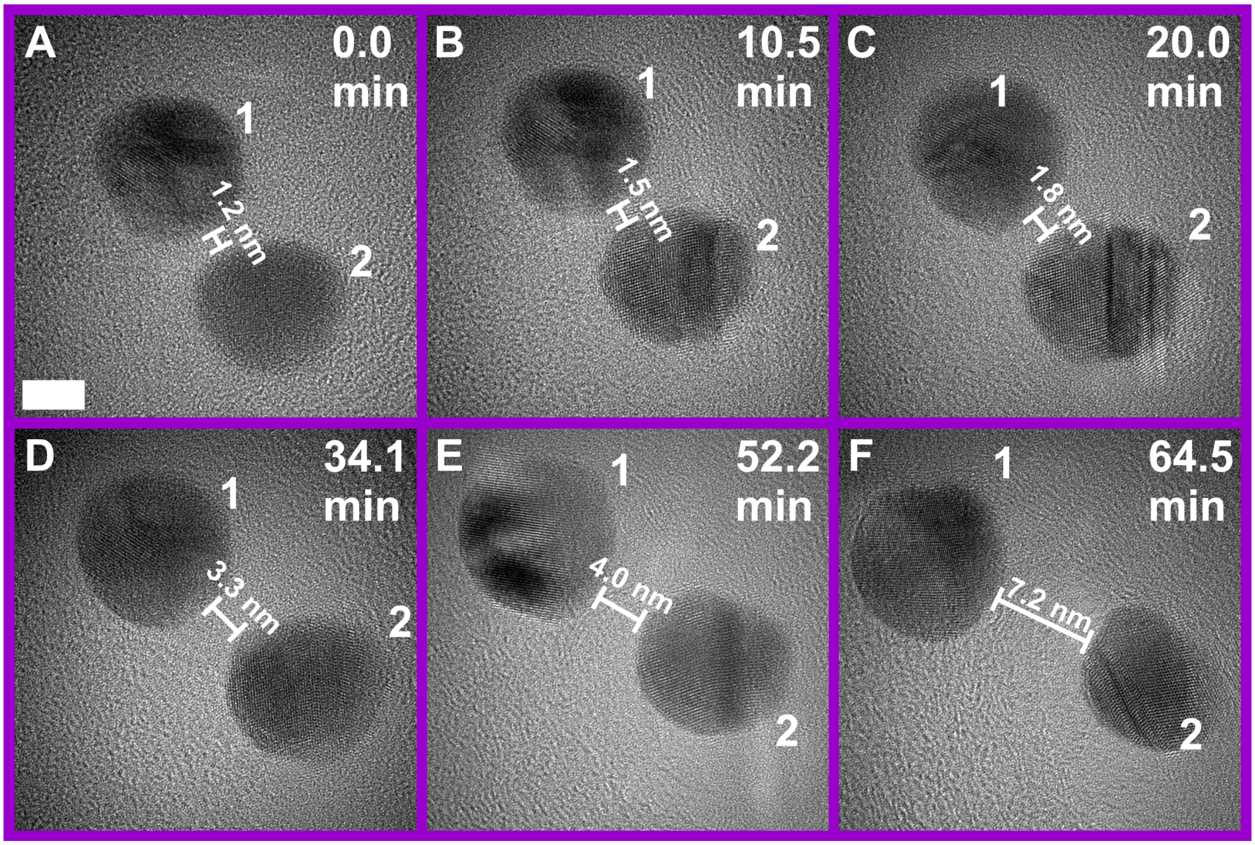

Figure S15. Repulsive character between a pair of large-sized AuNPs with *t* under electron beam exposure with D_1 and D_2 of 12.3 nm and 11.9 nm at 1.7×10^4 $e^-/Å^2s$ dose rate. The scale bar is 5 nm.

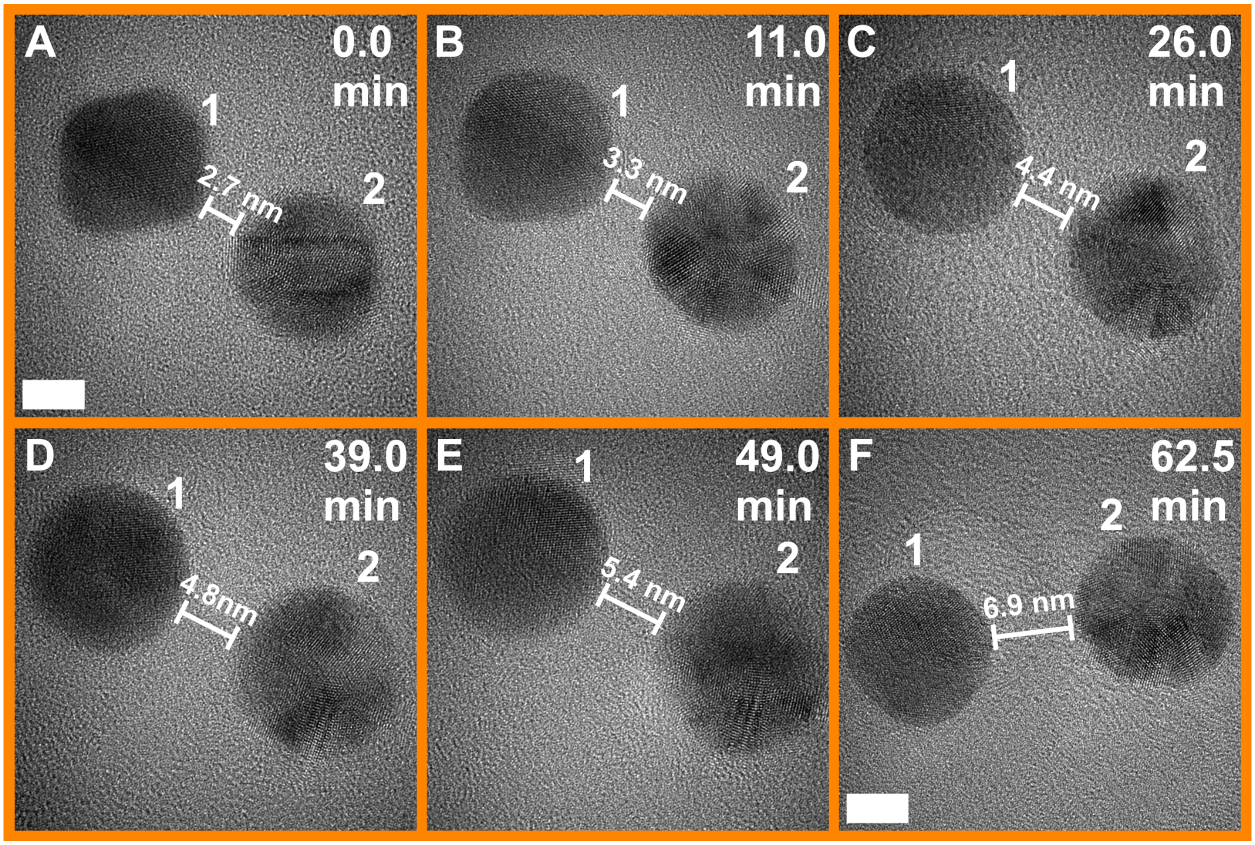

Figure S16. Repulsive character between a pair of large-sized AuNPs with t under electron beam exposure with D_1 and D_2 of 13.1 nm and 13.3 nm at 1.7×10^4 $e^-/Å^2s$ dose rate. The scale bar is 5 nm.

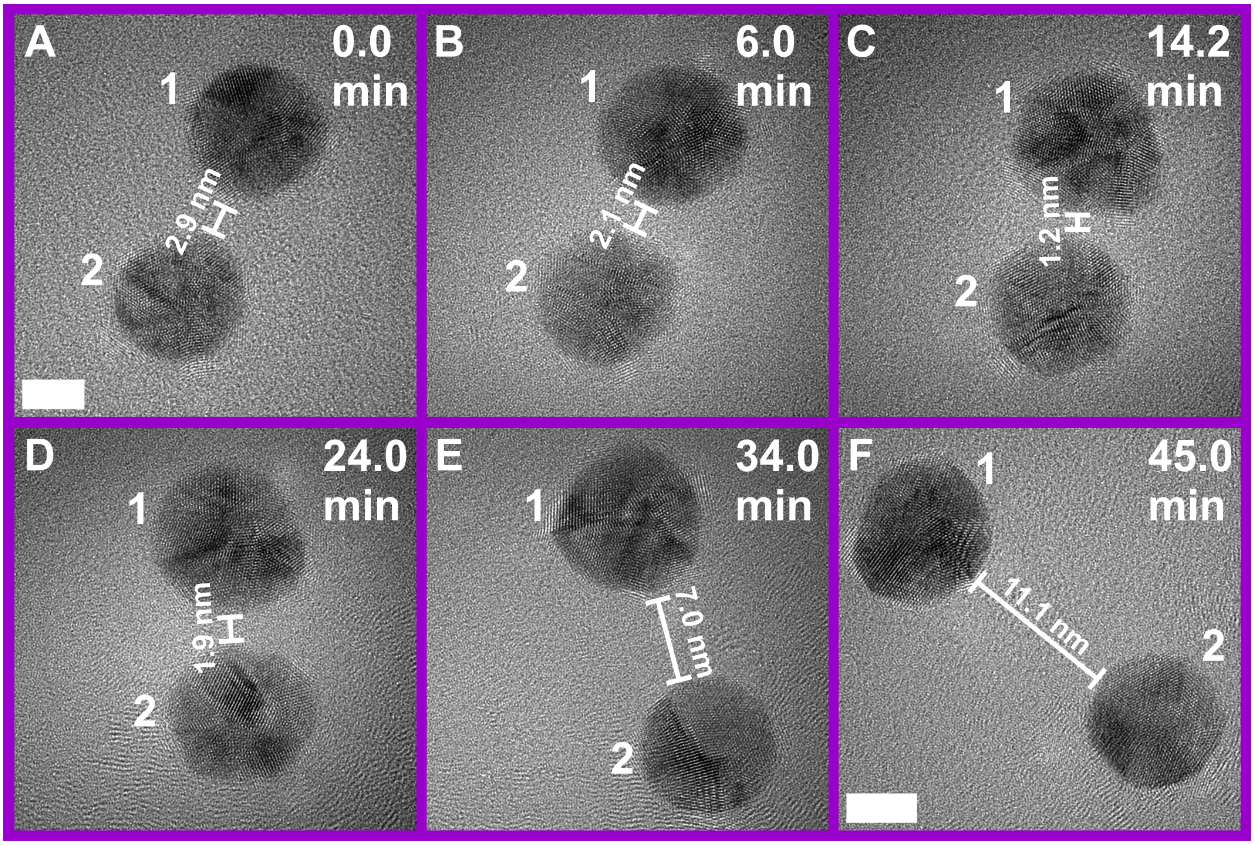

Figure S17. Repulsive character between a pair of large-sized AuNPs with t under electron beam exposure with D_1 and D_2 of 11.9 nm and 11.2 nm at 1.4×10^5 $e^-/Å^2s$ dose rate. The scale bar is 5 nm.

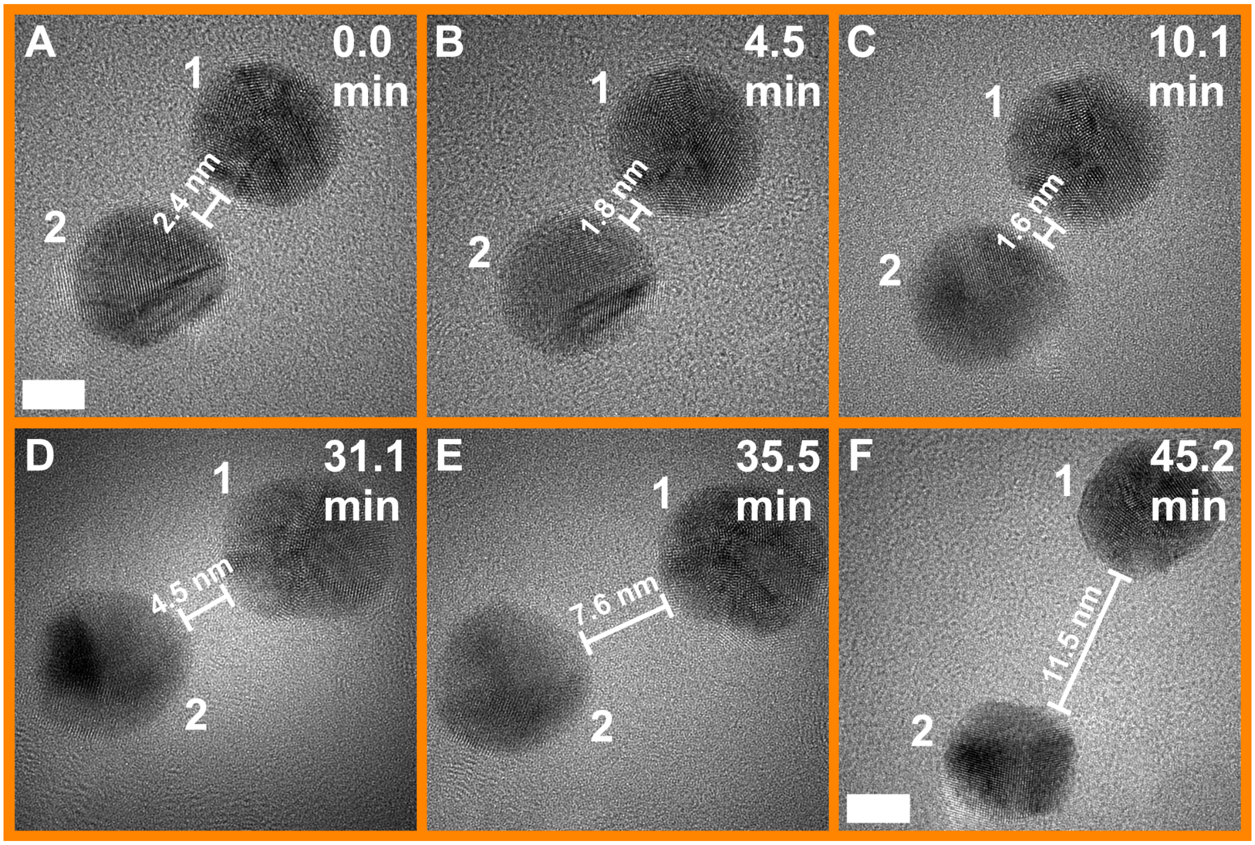

Figure S18. Repulsive character between a pair of large-sized AuNPs with *t* under electron beam exposure with D_1 and D_2 of 12.4 nm and 12.5 nm at 1.4×10^5 $e^-/Å^2s$ dose rate. The scale bar is 5 nm.

2.4. Coalescence in small and large-sized AuNPs

Section 2.4. in the main text.

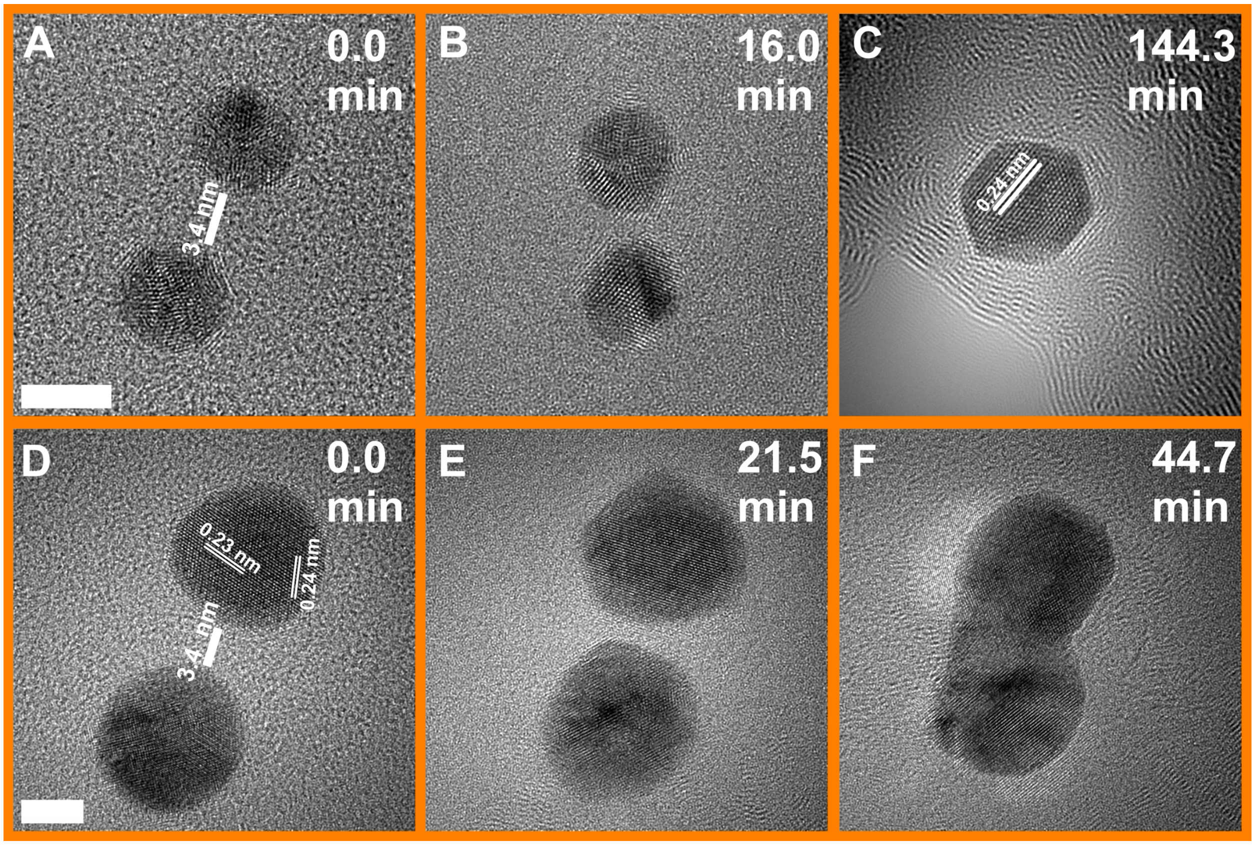

Figure. S19. A series of TEM micrographs showing the coalescence in small (A–C) and large-sized (D–F) AuNPs from a large distance.

3. Supplementary movies from in situ transmission electron microscopy imaging

Movie S1: Coalescence in small-sized gold (Au) nanoparticles (NPs) shown in Figure 3.

Movie S2: Growth of coalesced structure in small-sized AuNPs shown in Figure 6A, C and Figure S7.

Movie S3: Growth of coalesced structure in small-sized AuNPs shown in Figure 6B, D and Figure S8.

Movie S4: Growth of coalesced structure in large-sized AuNPs at low electron dose rate shown in Figure 6E, G and Figure S9.

Movie S5: Growth of coalesced structure in large-sized AuNPs at higher electron dose rate shown in Figure 6F, H and Figure S10.

Movie S6: Repulsion in small-sized AuNPs at low dose rate shown in purple panel in Figure 7A and Figure S11.

Movie S7: Repulsion in small-sized AuNPs at low dose rate shown in orange panel in Figure 7A and Figure S12.

Movie S8: Repulsion in small-sized AuNPs at higher dose rate shown in purple panel in Figure 7B and Figure S13.

Movie S9: Repulsion in small-sized AuNPs at higher dose rate shown in orange panel in Figure 7B and Figure S14.

Movie S10: Repulsion in large-sized AuNPs at low dose rate shown in purple panel in Figure 8A and Figure S15.

Movie S11: Repulsion in large-sized AuNPs at low dose rate shown in orange panel in Figure 8A and Figure S16.

Movie S12: Repulsion and attraction in large-sized AuNPs at higher dose rate shown in purple panel in Figure 8B and Figure S17.

Movie S13: Repulsion and attraction in large-sized AuNPs at higher dose rate shown in orange panel in Figure 8B and Figure S18.

Movie S14: Coalescence in small-sized AuNPs from a larger distance of 3.4 nm shown in Figure S19A–C.

Movie S15: Coalescence in large-sized AuNPs from a larger distance of 3.4 nm shown in Figure S19D–F and Figure S5.

Supplementary references

[1] H. Wu, H. Friedrich, J. P. Patterson, N. A. J. M. Sommerdijk, N. De Jonge, *Advanced Materials* **2020**, *32*, 2001582.
